# Structural Basis for EarP-Mediated Arginine Glycosylation of Translation Elongation Factor EF-P

**DOI:** 10.1128/mBio.01412-17

**Published:** 2017-09-26

**Authors:** Ralph Krafczyk, Jakub Macošek, Pravin Kumar Ankush Jagtap, Daniel Gast, Swetlana Wunder, Prithiba Mitra, Amit Kumar Jha, Jürgen Rohr, Anja Hoffmann-Röder, Kirsten Jung, Janosch Hennig, Jürgen Lassak

**Affiliations:** a Center for integrated Protein Science Munich (CiPSM), Department of Biology I, Microbiology, Ludwig-Maximilians-Universität München, Munich, Germany; b Structural and Computational Biology Unit, EMBL Heidelberg, Heidelberg, Germany; c Center for integrated Protein Science Munich (CiPSM), Department of Chemistry, Ludwig-Maximilians-Universität München, Munich, Germany; d University of Kentucky College of Pharmacy, Lexington, Kentucky, USA; Duke University School of Medicine

**Keywords:** *Pseudomonas aeruginosa*, *Pseudomonas putida*, TDP-rhamnose, glycosylation, glycosyltransferase, nucleotide sugar, posttranslational modification, ribosomes, translation

## Abstract

Glycosylation is a universal strategy to posttranslationally modify proteins. The recently discovered arginine rhamnosylation activates the polyproline-specific bacterial translation elongation factor EF-P. EF-P is rhamnosylated on arginine 32 by the glycosyltransferase EarP. However, the enzymatic mechanism remains elusive. In the present study, we solved the crystal structure of EarP from *Pseudomonas putida*. The enzyme is composed of two opposing domains with Rossmann folds, thus constituting a B pattern-type glycosyltransferase (GT-B). While dTDP-β-l-rhamnose is located within a highly conserved pocket of the C-domain, EarP recognizes the KOW-like N-domain of EF-P. Based on our data, we propose a structural model for arginine glycosylation by EarP. As EarP is essential for pathogenicity in *P. aeruginosa*, our study provides the basis for targeted inhibitor design.

## IMPORTANCE

The structural and biochemical characterization of the EF-P-specific rhamnosyltransferase EarP not only provides the first molecular insights into arginine glycosylation but also lays the basis for targeted-inhibitor design against *Pseudomonas aeruginosa* infection.

## INTRODUCTION

**FIG 1:**
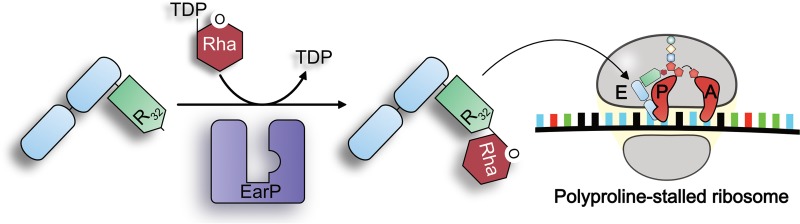
Activation and molecular function of EarP-arginine-type translation elongation factor EF-P. (Left) The bacterial translation elongation factor EF-P is composed of two OB-Fold domains (light blue) and one KOW-like N-domain (light green). In about 10% of all bacteria, EF-P is posttranslationally activated by α-glycosylation of a strictly conserved arginine (R32) (17, 26). The glycosylation reaction is catalyzed by the EF-P–arginine rhamnosyltransferase EarP (purple) using dTDP-β-l-rhamnose (TDP-Rha [red]) as a substrate. (Right) Activated EF-P is recruited to polyproline-stalled ribosomes and binds between the E and P sites. Thereby, R32^EF-P^ and the attached rhamnose moiety presumably stabilize the CCA end of the P-site prolyl-tRNA, which in turn stimulates Pro–Pro peptide bond formation and thus alleviates the translational arrest.

Translation elongation is a nonuniform process and directly depends on the amino acids (aa) to be incorporated into the growing polypeptide chain (1). Due to its chemical and physical properties, proline delays the peptidyl transfer reaction (2), and ribosomes can even stall upon translation of distinct diprolyl-containing sequence motifs (Fig. 1) (3, 4). Such ribosome stalling is alleviated by the eukaryotic and archaeal elongation factor 5A (e/aEF-5A) (5–7) and its prokaryotic orthologue the bacterial translation elongation factor P (EF-P) (8–14). The L-shaped EF-P is composed of three β-barrel domains and structurally resembles tRNA in both size and shape (15). EF-P binds to the polyproline-stalled ribosomes between the binding sites of peptidyl-tRNA (P-site) and the exiting tRNA (E-site) (16) and stimulates peptide bond formation by stabilization of the CCA end of the P-site prolyl-tRNA (Fig. 1) (17, 18). A conserved positively charged residue—located at the tip of the EF-P KOW-like N-domain—is essential for function (11, 17). However, for full EF-P activity, this residue is posttranslationally elongated (19). Certain bacteria—including *Escherichia coli* and *Salmonella enterica*—ß-lysinylate a conserved lysine, K34^EF-P^, by EpmA. This EF-P-specific ligase uses β-(*R*)-lysine as the substrate, which is generated by isomerization of α-(*S*)-lysine by employing the activity of the amino mutase EpmB (20–23). In contrast, activation of a phylogenetically distinct group of EF-Ps encoded in species such as *Pseudomonas aeruginosa* and *Neisseria meningitidis* depends on rhamnosylation of an arginine, R32^EF-P^, in the equivalent position (17, 24, 25). Rhamnosylation is mediated by the recently discovered inverting glycosyltransferase EarP, which utilizes dTDP-beta-l-rhamnose (TDP-Rha) as donor substrate, resulting in α-rhamnosyl-arginine on the acceptor EF-P (26, 27). Unlike with the common and relatively well understood glycosylation of asparagine, sugar modifications on the guanidino group of arginine appear to be rare, and almost nothing is known about the molecular mechanism (28, 29). To date, there are only two reported cases of arginine glycosylation other than EF-P rhamnosylation. The first one described self-β-glycosylation of sweet corn amylogenin (30). In the second case, an effector glycosyltransferase termed NleB of enteropathogenic *E. coli* (EPEC) was shown to inactivate human cell death domain-containing proteins by *N*-acetylglucosaminylation of arginine, with this being a major pathogenicity determinant during infection (31). Similarly, a lack of *earP* abolishes the pathogenicity of *P. aeruginosa* (17). Accordingly, solving the molecular mechanism of arginine rhamnosylation might pave the way to ultimately design and develop targeted inhibitors against EarP.

Here we present the X-ray crystal structure of EarP from *Pseudomonas putida* KT2440 (EarP_*Ppu*_) bound to its cognate nucleotide-sugar donor substrate TDP-Rha at a 2.3-Å resolution (PDB accession number 5NV8). Together with reporting the results of nuclear magnetic resonance (NMR) spectroscopy analyses and an *in vitro*/*in vivo* biochemical enzyme characterization, we lay the foundation for understanding arginine glycosylation.

## RESULTS

Despite low sequence conservation most nucleotide sugar dependent (Leloir-type) glycosyltransferases adopt one of two major folding patterns, GT-A or GT-B (28). However, so far, there is no available information on the structure and folding properties of EarP. We used SWISS-MODEL (32), Phyre^2^ (33), and the I-TASSER server for protein structure and function predictions (34–36) to generate fold recognition models of EarP from *Pseudomonas putida* (see Fig. S3A in the supplemental material). These predictions suggested the UDP-*N*-acetylglucosamine (UDP-GlcNAc)-dependent glycosyltransferases MurG from *E. coli* (MurG_*Eco*_) (37) and O-GlcNAc transferase (OGT) from *Xanthomonas campestris* (OGT_*Xca*_) (38) as structural orthologues. Accordingly, EarP_*Ppu*_ adopts a clamp-like structure with two opposing Rossmann-like domains that are separated by an interdomain cleft (Fig. S3A). With this, the protein is presumably a GT-B-type glycosyltransferase (28).

10.1128/mBio.01412-17.1 FIG S1  (A) Specificity of anti-Arg^Rha^ antibodies. One point five micrograms of unmodified EF-P (EF-P^−^) and 0.5 µg modified EF-P (EF-P^Rha^) were subjected to SDS-PAGE and transferred to a nitrocellulose membrane by horizontal Western blotting. The membrane was cut, and the halves were used to detect EF-P_*Ppu*_ using 0.2 µg/ml anti-EF-P and EF-P^Rha^ using 0.25 µg/ml anti-Arg^Rha^ antibodies. (B) *In vivo* functionality analysis of EarP_*Ppu*_ single-amino-acid exchange variants. (Top) β-Galactosidase activity in *E. coli* MG1655 P_*cadBA*_::*lacZ* Δ*efp* upon expression of EF-P_*Ppu*_ and EarP_*Ppu*_ and coexpression of EF-P_*Ppu*_ with EarP_*Ppu*_ and single-amino-acid substitution variants. Cells were incubated under *cadBA*-inducing conditions (LB, pH 5.8) at 30°C o/n (17). Data are mean values from three independent replicates ± standard deviations. Asterisks indicate significance. (Middle) The presence of heterologously expressed His_6_-tagged EF-P_*Ppu*_ was verified using 0.1 µg/ml anti-His_6_ tag (Abcam, Inc.). (Bottom) Rhamnosylation of EF-P_*Ppu*_ in MG1655 cells coexpressing EF-P_*Ppu*_ and its EarP_*Ppu*_ single-amino-acid exchange variants was assessed by Western blotting and detection of EF-P^Rha^ using 0.25 µg/ml anti-Arg^Rha^. Download FIG S1, TIF file, 1.8 MB. Copyright © 2017 Krafczyk et al. 2017 Krafczyk et al. This content is distributed under the terms of the Creative Commons Attribution 4.0 International license.

10.1128/mBio.01412-17.2 FIG S2  (A) Time-dependent *in vitro* rhamnosylation of EF-P_*Ppu*_ by EarP_*Ppu*_. Fixed amounts of EF-P_*Ppu*_ (2.5 µM), EarP_*Ppu*_ (0.1 µM), and TDP-Rha (500 µM) were incubated at 30°C in 100 mM NaP_i_, pH 7.6, for various amounts of time. Measurements were performed in technical duplicates. Standard errors are shown. A representative SDS-PAGE gel and Western blot used for generation of the time course curve are shown. Rhamnosylated EF-P_*Ppu*_ (EF-P^Rha^) was detected using 0.25 µg/ml anti-Arg^Rha^. Band intensities were quantified using ImageJ (76). (B) TDP-Rha saturation curves of EarP_Ppu_ single-amino-acid exchange variants. Fixed amounts of EF-P_*Ppu*_ (2.5 µM) and the respective EarP_*Ppu*_ single-amino-acid exchange variants (0.1 µM) were incubated with various concentrations of TDP-Rha at 30°C in 100 mM NaP_i_, pH 7.6. In the cases of the F191A^EarP^ and Y1931A^EarP^ variants, 0.5 µM concentrations were used. Suitable incubation times were determined by time course experiments prior to determination of kinetic parameters (data not shown). Representative SDS-PAGE gels and Western blots used for generation of the TDP-Rha saturation curves are depicted underneath the corresponding graphs. Rhamnosylated EF-P_*Ppu*_ was detected after Western blotting using 0.25 µg/ml anti-Arg^Rha^. Band intensities were quantified using ImageJ (76). *K*_*m*_ and *k*_cat_ were calculated using SigmaPlot. Standard errors are shown when measurements were performed in technical duplicates. Download FIG S2, TIF file, 3.6 MB. Copyright © 2017 Krafczyk et al. 2017 Krafczyk et al. This content is distributed under the terms of the Creative Commons Attribution 4.0 International license.

10.1128/mBio.01412-17.3 FIG S3  (A) Fold recognition models of EarP from *P. putida*. (Top) Structural models of EarP from *P. putida* were generated using Phyre^2^ (orange), the I-TASSER server (white), and SWISS-MODEL (blue). Model coverage and scoring is shown underneath the respective model structures. (Bottom) Overlay of structural models from Phyre^2^ and I-TASSER (left), I-TASSER and SWISS-MODEL (middle), and SWISS-MODEL and Phyre^2^ (right). Root mean square deviations of atomic positions are shown underneath the respective structural overlays. All illustrations and overlays were generated using UCSF Chimera and the UCSF Chimera MatchMaker (82). (B) B-factors are plotted on the crystal structure of EarP. Several regions in the N-domain show significantly higher B-factors than in the C domain, suggesting the mobile nature of this region. (C) Electron density of TDP-Rha in the donor binding pocket. TDP-Rha is depicted as sticks. Sigma-Aldrich weighted 2mFo-DFc map of TDP-Rha contoured at 1 *σ* is depicted as grey mesh. (D) SAXS analysis of free EarP and EarP bound to TDP-Rha. The two experimental small-angle X-ray scattering curves of free EarP (black) and EarP plus TDP-Rha (blue) do not exhibit any significant changes. Thus, it can be concluded that there are no major conformational changes upon ligand binding. The crystal structure and monomeric states of the protein are also validated in solution, as the back-calculated scattering densities (red line) fit well with the experimental data (χ^2^ = 1.96). The deviation at the low Q range is due to aggregation and/or interparticle interaction. (E) Secondary structure of EF-P from *P. putida*. The secondary structures of individual amino acids are indicated as a propensity to form either α-helix (red) or β-strand (green). The amino acids with a propensity to adopt a random coil or lacking information about secondary structure were assigned zero values in the plot. The propensity values were obtained from C_α_, C_β_, and C′ secondary chemical shifts using CcpNmr Analysis (106). (F) Secondary structure of EF-P from *P. aeruginosa* (PDB accession number 3OYY) (45). Red ribbons indicate α-helices; green arrows indicate β-strands. Download FIG S3, TIF file, 10.7 MB. Copyright © 2017 Krafczyk et al. 2017 Krafczyk et al. This content is distributed under the terms of the Creative Commons Attribution 4.0 International license.

### Structure of *Pseudomonas putida* EarP.

**FIG 2:**
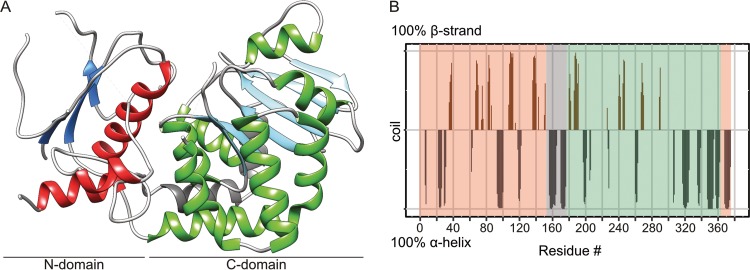
EarP folding pattern and topology. (A) Ribbon representation of the 2.3-Å crystal structure of EarP_*Ppu*_. The illustration was generated with UCSF Chimera (82). Secondary-structure elements are shown, with α-helices in red and green for the N- and C-domains, respectively, and β-strands correspondingly in blue and cyan. The bipartite helix of the linker domain is grey. β-Strand 3, α-helix 5, and short loops with weak electron density are also displayed here but are missing in the PDB coordinate file to improve statistics, as discontinuity in the electron density did not allow proper modeling. (B) Secondary structure of EarP determined by NMR secondary shifts. The secondary structure of individual amino acids is indicated as propensity to form either an α-helix (grey) or a β-strand (brown). The amino acids with a propensity to adopt a random coil or lacking information about secondary structure were assigned zero values in the plot. The propensity values were obtained from C_α_, C_β_, NH, and H chemical shifts using TALOS+ (103). The N- and C-terminal EarP domains are boxed in peach and green, respectively. The interconnecting linker is boxed in grey.

We were able to subsequently confirm the GT-B fold by having solved the crystal structure of EarP_*Ppu*_ at 2.3-Å resolution (Fig. 2A; Data Set S2). Indeed, the EarP_*Ppu*_ C-domain includes residues 184 to 361 and follows the Rossmann fold topology, with six β-strands (β8 to β13) and seven α-helices (α8 to α14) (Fig. 2 and see Fig. 4A). On the other hand, the N-domain (aa 1 to 153 and 362 to 377) could only be built in part. Although weak electron density for likely other regions of the N-domain was noticed, it was not sufficient to be unambiguously and reliably interpreted as particular missing parts of the protein chain. It is important to note that there is no indication that the diffraction data are twinned or anisotropic. The poor density of the N-domain is not caused by misinterpretation of noncrystallographic symmetry as crystallographic symmetry, because choosing a space group with lower symmetry does not improve the electron density. Yet, the structure has a higher-than-usual R-free (35.1%) value at this resolution, which cannot be explained by a simple absence of disordered regions. This is likely due to the N-domain adopting different conformations in different unit cells, causing crystal disorder. The potential mobility of the N-domain is further supported by higher average B-factors for this domain than for the C-domain (61 Å^2^ versus 46 Å^2^) (see Fig. S3B for B-factors mapped onto the protein structure). In addition, our rigorous attempts to obtain crystals in different space groups by screening various crystallization conditions were not successful. In the predicted structure (Fig. S3A, model 2), the N-domain features a central β-sheet of seven β-strands (β1 to β7), surrounded by the α-helices (α1 to α5 and α15) (see Fig. 4A and Fig. S3A). In the crystal structure, only β-strands β1, β2, and β3, as well as α-helixes α1, α5, and α15, are modeled (Fig. 2A). However, the missing structural elements in the protein N-domain are not in close vicinity to the active site according to the fold recognition model (Fig. S3A, model 2), and we did not observe any unassigned electron density in the vicinity of the ligand. Thus, despite this disorder, our crystal structure still provides crucial information important for understanding ligand binding and the catalytic mechanism. For structure validity assessment, the EarP crystal structure with electron density is shown in Fig. S4 in the supplemental material.

10.1128/mBio.01412-17.8 DATA SET S1  Strains, plasmids, and primers used in the study. Download DATA SET S1, XLSX file, 0.02 MB. Copyright © 2017 Krafczyk et al. 2017 Krafczyk et al. This content is distributed under the terms of the Creative Commons Attribution 4.0 International license.

10.1128/mBio.01412-17.9 DATA SET S2  Crystallographic data collection and refinement statistics. Download DATA SET S2, XLSX file, 0.01 MB. Copyright © 2017 Krafczyk et al. 2017 Krafczyk et al. This content is distributed under the terms of the Creative Commons Attribution 4.0 International license.

10.1128/mBio.01412-17.4 FIG S4  (A) Stereo view of the EarP–TDP-Rha complex. Electron density is shown. (B and C) Electron densities for the N- and C-terminal domains, respectively. The N-terminal domain shows several disconnected blobs of electron density currently modeled with water, and some regions are entirely missing. Download FIG S4, TIF file, 7.9 MB. Copyright © 2017 Krafczyk et al. 2017 Krafczyk et al. This content is distributed under the terms of the Creative Commons Attribution 4.0 International license.

Furthermore, the presence of the predicted strands and helices and thus the validity of the model and crystal structure could be confirmed by NMR secondary chemical shifts (Fig. 2B). A prerequisite for this analysis is the backbone chemical shift assignment by triple-resonance NMR experiments. The relatively large size of EarP_*Ppu*_ at 43 kDa exceeds the sensitivity limitations of NMR, demanding deuteration in order to decrease cross-relaxation effects and to decrease the signal line width. Nonetheless, using transverse relaxation-optimized spectroscopy (TROSY)-based experiments, we were able to assign 62% of the EarP_*Ppu*_ backbone.

The two domains are interconnected by a bipartite helix (α6, α7) comprising aa 156 to 176. This linker region together with an unstructured segment that positions α15 in the vicinity of the N terminus defines the floor of the cleft that separates the domains (Fig. 2A and see Fig. 4).

Based on these and previous data (17, 24–27), EarP was built in the carbohydrate-active enzymes (CAZy) database (39) and now represents the new glycosyltransferase family GT104.

### Analysis of the TDP-β-l-rhamnose binding site in the EarP C-domain.

**FIG 3:**
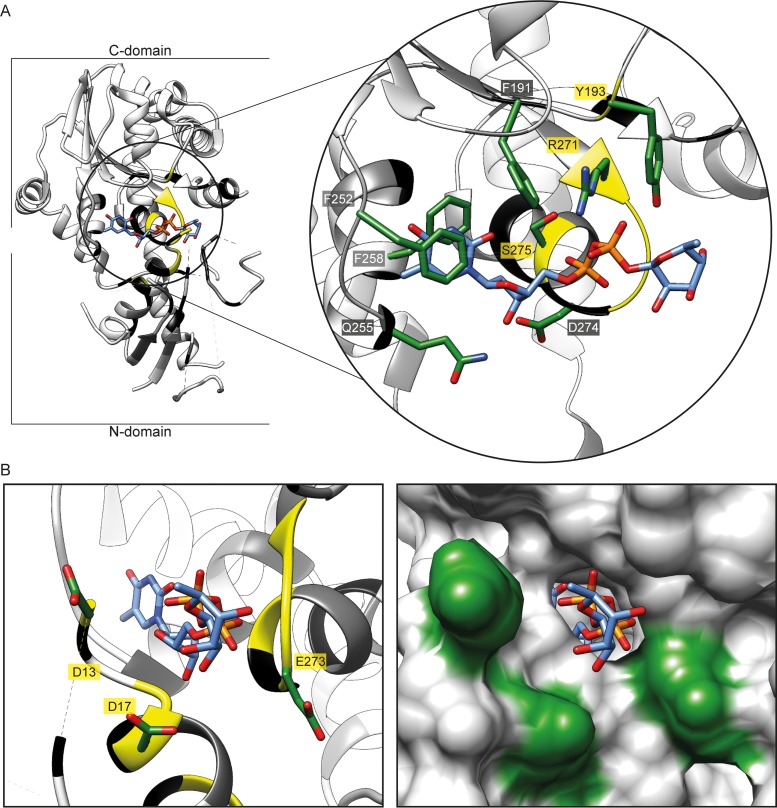
EarP TDP-β-l-rhamnose binding site. (A, left) Three-dimensional structure of EarP_*Ppu*_ in a ribbon representation. The TDP-Rha binding pocket in the C-domain is circled in black. (Right) Zoom into the nucleotide-sugar binding pocket with bound TDP-Rha (blue sticks). Important residues for TDP-Rha positioning are depicted as green sticks and labeled with single-letter code identifiers. (B, left) Ribbon representation of the nucleotide-sugar binding pocket with stick representation of bound TDP-Rha (blue sticks) as well as the three invariant residues D13, D17, and E273 (green sticks), which are presumably involved in catalysis. (Right) Surface representation of the nucleotide-sugar binding pocket with stick representation of bound TDP-Rha (blue). Surfaces of D13, D17, and E273 are in green. Ribbons are color coded according to their degree of conservation, as follows: yellow, 100%; black, ≥95%; dark grey, ≥90%; light grey, ≥50%; and white, <50% identical residues in all analyzed EarP orthologues. The electron density for TDP-Rha bound to EarP is shown in Fig. S3C. All illustrations were generated with UCSF Chimera (82).

In Leloir-type GT-B glycosyltransferases, the nucleotide-sugar binding site is canonically located in the protein C-domain (40). Similarly, TDP-Rha in the EarP_*Ppu*_ crystal structure is located in a binding pocket that is composed of residues located in the C-domain (Fig. 3A). F191^EarP^, F252^EarP^, and F258^EarP^ side chains form an aromatic cage that stacks against the base of the nucleotide moiety of TDP-Rha. The sugar ring of the nucleotide is then specifically recognized by a hydrogen bond between the hydroxyl group on C3′ of the sugar and the side chain of Q255^EarP^. The diphosphate is recognized by hydrogen bonds formed with the side chain guanidine of R271^EarP^, the Y193^EarP^ side chain hydroxyl, and backbone amides of E273^EarP^ and D274^EarP^. The binding pocket is closed by the bulky side chain of Y193^EarP^, which may sterically ensure proper positioning of the rhamnose sugar (Fig. 3A). The rhamnose sugar itself does not seem to make any contact with the protein and is solvent exposed. We further confirmed this by saturation transfer difference (STD) NMR experiments (41), where we did not observe any difference signal from the rhamnose moiety but did observe one from the TDP moiety of TDP-Rha (Fig. S5A).

10.1128/mBio.01412-17.5 FIG S5  (A) STD NMR at a 1:100 ratio of protein to ligand shows several signals from the TDP but no signal from the sugar region of the TDP-Rha, thus confirming the crystal structure where the sugar is solvent exposed, and does not show extensive contacts with the protein. (B) STD NMR with a low excess of TDP-Rha (1:7 ratio of protein to ligand) clearly shows the signal for the methyl group of the thymidine ring, confirming the interaction between TDP-Rha and EarP under conditions used for SAXS data collection. (C) STD NMR experiments with E273 variants showing that the mutation of E273 to alanine, glutamine, or aspartate does not affect the binding of TDP-Rha to EarP. Download FIG S5, TIF file, 3.2 MB. Copyright © 2017 Krafczyk et al. 2017 Krafczyk et al. This content is distributed under the terms of the Creative Commons Attribution 4.0 International license.

In parallel, small-angle X-ray scattering (SAXS) of free EarP_*Ppu*_ and EarP_*Ppu*_ bound to TDP-Rha has been performed (Fig. S3D). The overall shape of the molecule could be validated to be the same in solution. Protein backbone conformational changes upon TDP-Rha binding are confirmed by chemical shift perturbations (see Fig. 7B); however, SAXS indicates that there are no large (>10-Å) conformational changes or movements of the two Rossmann fold domains with respect to each other upon binding of TDP-Rha, as the scattering density does not change from that in the free state. To show that TDP-Rha is bound to EarP under SAXS experimental conditions, STD NMR experiments were performed. They confirm again that TDP-Rha binding occurs with the ligand at a 7-fold excess compared to the amount of protein (Fig. S5B).

Database mining identified 432 EarP homologues representing about 10% of sequenced bacteria (Data Set S3) (17). Phylogenetically, EarP originated presumably in the betaproteobacterial subdivision and was horizontally transferred into the gammaproteobacterial orders of *Pseudomonadales*, *Aeromonadales*, and *Alteromonadales* (17). It can also be found in certain *Fusobacteria*, *Planctomycetes*, and *Spirochetes* (17).

10.1128/mBio.01412-17.10 DATA SET S3  Collection of EarP homologues. Download DATA SET S3, XLSX file, 0.1 MB. Copyright © 2017 Krafczyk et al. 2017 Krafczyk et al. This content is distributed under the terms of the Creative Commons Attribution 4.0 International license.

**FIG 4:**
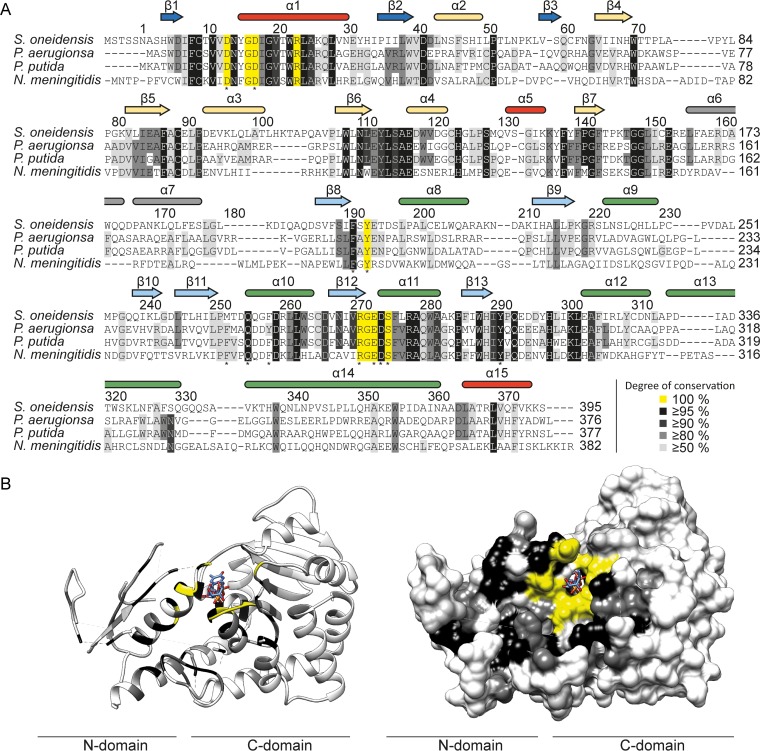
Evolutionary conservation of amino acids in EarP homologues. (A) Multiple-sequence alignment of EarP proteins from *Shewanella oneidensis*, *P. aeruginosa*, *P. putida*, and *Neisseria meningitidis* as a selection from the alignment of 432 protein sequences that were collected from the NCBI database (Data Set S3). The multiple-sequence alignment was generated using Clustal Omega (104). Secondary-structure elements of EarP are shown and based on the EarP_*Ppu*_ crystal structure, NMR analysis, and predictions by MINNOU (105). α-Helices are in red and green for the N- and C-domains, respectively, and β-strands are in blue and cyan. The bipartite helix of the linker domain is grey. Helices and β-strands not resolved in the crystal structure are yellow. Amino acids selected for mutational analysis are indicated by asterisks. (B) The EarP_*Ppu*_ crystal structure was colored according to the degree of conservation of the respective amino acids. Ribbon (left) and surface (right) representations of the EarP_*Ppu*_ crystal structure are shown. Colors indicate the following: yellow, 100%; black, ≥95%; dark grey, ≥90%; light grey, ≥50%; and white, <50% identical residues in all analyzed EarP orthologues. Illustrations were generated with UCSF Chimera (82).

In order to identify conserved amino acids, we used Clustal Omega (42) and generated a multiple-sequence alignment (Fig. 4A). We found 49 residues with a sequence conservation of ≥95%. Mapping of these residues onto the crystal structure revealed an accumulation at or near the interdomain cleft (Fig. 4B), including the binding pocket for the nucleotide sugar donor substrate (Fig. 3A), which is highly supportive of the correctness of the solved structure.

**FIG 5:**
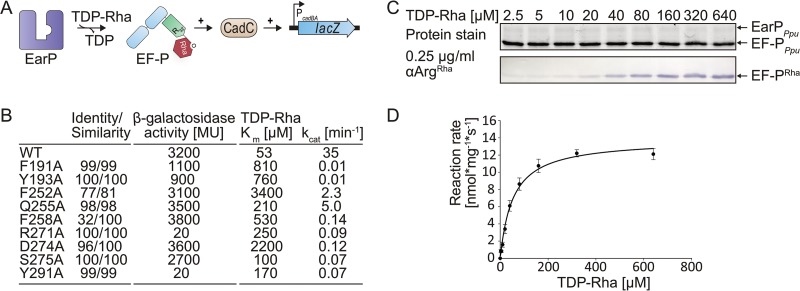
Analysis of kinetic parameters and *in vivo* activities of EarP_*Ppu*_ variants. (A) Molecular principle of the *in vivo* EF-P_*Ppu*_ functionality assay. This assay is based on the lysine decarboxylase acid stress response of *E. coli*, the CadABC module (68). At low pH and with the concomitant presence of lysine, the transcriptional activator CadC activates the promoter of its two downstream genes (P_*cadBA*_) and with this induces expression of *lacZ* in an *E. coli* MG1655 P_*cadBA*_::*lacZ* strain (11). Proper translation of CadC is dependent on the presence of EF-P and its corresponding modification system, and thus β-galactosidase activity can be taken as a direct readout for EF-P and EarP functionality. (B) Degree of conservation (identity/similarity) in percent, *in vivo* activities, and kinetic parameters of tested single-amino-acid exchange variants of EarP_*Ppu*_. *In vivo* EarP_*Ppu*_ activities were determined by measuring the β-galactosidase activities of an *E. coli* MG1655 P_*cadBA*_::*lacZ* Δ*efp* strain heterologously expressing *efp*_*Ppu*_ together with wild-type or mutant *earP*_*Ppu*_ genes from o/n cultures in LB (pH 5.8). Background corrected mean values from three independent measurements are shown. Standard deviations were determined from three independent experiments to be ≤10%; the *K*_*m*_ and *k*_cat_ of wild-type EarP_*Ppu*_ (WT^EarP^) and variants with single-amino-acid substitutions are given in micromolar concentrations and per minute, respectively. Standard errors were determined by SigmaPlot to be <20%. (C, top) 2,2,2-Trichlorethanol (TCE) protein stain (75) of a representative SDS gel used for determination of kinetic parameters. Fixed amounts of EF-P_*Ppu*_ (2.5 µM) and WT^EarP^ (0.1 µM) were incubated with various concentrations of TDP-Rha for 20 s and subjected to SDS-PAGE. (Bottom) Detection of rhamnosylated EF-P_*Ppu*_. EF-P_*Ppu*_ was visualized after Western blotting using 0.25 µg/ml anti-Arg^Rha^. (D) TDP-Rha saturation curve of WT^EarP^. Band intensities from panel C were quantified using ImageJ (76). Reaction rates were calculated as means of four independent measurements. Standard deviations are shown as error bars for each concentration.

To substantiate our structural findings with biochemical data, we prepared EarP_*Ppu*_ constructs with single-amino-acid substitutions of the individual residues forming the binding pocket and tested the activities of the EarP_*Ppu*_ variants both *in vivo* and *in vitro* (Fig. 5). This included F191^EarP^, F252^EarP^, and F258^EarP^, which form the aromatic pocket, as well as Y193^EarP^, Q255^EarP^, R271^EarP^, and D274^EarP^, which are involved in hydrogen bond networking (Fig. 5B).

Previously, we could show that the heterologous expression of *efp* and *earP* from *Shewanella oneidensis* in *E. coli* can fully complement a lack of EF-P (17) with respect to the activation of the lysine-dependent acid stress response by the transcriptional activator CadC (11). Similarly, coproduction of wild-type EF-P_*Ppu*_ and wild-type EarP_*Ppu*_ (WT^EarP^) can restore β-galactosidase activity in an *E. coli* P_*cadBA*_::*lacZ* Δ*efp* strain (Fig. 5A and S1B). From the nine tested EarP_*Ppu*_ substitution variants, we measured reduced β-galactosidase activities for the variants F191A^EarP^, Y193A^EarP^, R271A^EarP^, S275A^EarP^, and Y291A^EarP^. The variants R271A^EarP^ and Y291A^EarP^ failed to induce β-galactosidase expression at all (Fig. 5B and S1B).

In parallel, the enzymatic activity of EarP_*Ppu*_ was investigated *in vitro* by employing an anti-Arg^Rha^ antibody. The antibody was raised against a chemically synthesized glycopeptide antigen (SGR^Rha^NAAIVK) and specifically detects arginine rhamnosylation (see Materials and Methods) (Fig. S1A). This in turn enabled the quantification of rhamnosylation rates of EF-P_*Ppu*_ by Western blot analysis (Fig. 5C and D). In a first step, the *K*_*m*_ and *k*_cat_ of WT^EarP^ were determined to be 53 µM and 35 min^−1^, respectively (Fig. 5B, C, and D).

We wondered whether this *K*_*m*_ makes sense physiologically and therefore analyzed the cellular TDP-Rha levels in *P. putida*, *P. aeruginosa*, and *E. coli*, which were 3.5 mM, 2.0 mM, and 4.0 mM, respectively (see Materials and Methods and Fig. S6). In good accordance with our measurements, the physiological TDP-Rha concentration in *Lactococcus lactis* was previously determined to be as high as 1 mM (43). Thus, within a bacterial cell, the donor substrate reaches saturating concentrations, according to the WT^EarP^ *K*_*m*_ measurements.

10.1128/mBio.01412-17.6 FIG S6  (A) SDS-PAGE gels and Western blots for determination of the intracellular TDP-Rha concentration in *E. coli* MG1655 (top), *P. putida* KT2440 (middle), and *P. aeruginosa* (bottom). Purified EF-P_*Ppu*_ (2.5 µM) and EarP_*Ppu*_ (0.1 µM) were incubated with increasing concentrations of TDP-Rha or lysates from ~10^7^ and ~10^8^ cells (sample volume, 20 µl; 100 mM NaP_i_ [pH 7.6]; 20 s; 30°C). Samples containing cell lysates were incubated in the presence and absence of EarP_*Ppu*_. Rhamnosylated EF-P (EF-P^Rha^) was detected using 0.25 µg/ml anti-Arg^Rha^. For *E. coli* and *P. aeruginosa*, experiments were performed as biological triplicates. (B) Representative calibration curves calculated from relative Western blot band intensities in samples containing increasing TDP-Rha concentrations. Band intensities were quantified using ImageJ (76). TDP-Rha concentrations in samples containing ~10^8^ lysed cells (triangles) were calculated from the slope of the calibration curve and are indicated by dashed lines. Band intensities from samples containing no EarP_*Ppu*_ were subtracted from those containing EarP_*Ppu*_ to correct for background signal. (C) Equations used to calculate average TDP-Rha concentrations in single cells. For calculation of average TDP-Rha concentrations in *E. coli* cells, an average cell volume of 3.9 fl was assumed (84). For calculation of average TDP-Rha concentrations in *P. putida* and *P. aeruginosa* cells, an average cell volume of 2.1 fl was assumed (85). Download FIG S6, TIF file, 6.9 MB. Copyright © 2017 Krafczyk et al. 2017 Krafczyk et al. This content is distributed under the terms of the Creative Commons Attribution 4.0 International license.

Next, the *K*_*m*_ and *k*_cat_ of EarP_*Ppu*_ substitution variants were determined and compared to those of the wild-type protein. Strikingly, all *earP* mutations affected enzymatic activity (Fig. 5B and S2B). Depending on the substituted residue, the *K*_*m*_ increased up to 60-fold for the F252A^EarP^ variant (*K*_*m*_ = 3.4 mM). Conversely, the *k*_cat_ decreased up to 3,500 times when we measured the kinetics of the F191A^EarP^ and Y193A^EarP^ variants.

To exclude the possibility that decreased enzyme activity was due to fold disruption, selected EarP_*Ppu*_ variants (F191A^EarP^, Y193A^EarP^, F252A^EarP^, R271A^EarP^, D274A^EarP^, and Y291A^EarP^) were analyzed by NMR ^1^H-^15^N heteronuclear single quantum coherence (HSQC) experiments (Fig. S7). All tested substitution variants showed no structural alterations from the wild-type protein, except for the D274A^EarP^ variant. The structural instability of this EarP variant might be a result of disrupting a salt bridge that is formed between the side chains of D274^EarP^ in the protein C-domain and an equally conserved arginine at position 23 (R23^EarP^) in the protein N-domain (Fig. 4). This salt bridge might be of importance in clamping both EarP domains together, and a lack of it might therefore destabilize the protein. Indeed, further purification of the D274A^EarP^ variant by size exclusion chromatography (SEC) revealed an elution pattern with three distinct EarP peaks, indicating a certain degree of protein aggregation. However, the lowest molecular peak in the D274A^EarP^ SEC profile is congruent with the one that we found when subjecting WT^EarP^ to SEC. Accordingly, *K*_*m*_ (TDP-Rha) and *k*_cat_ values were determined from this protein fraction to be 206 µM and 0.74 min^−1^, respectively (Fig. 5B).

10.1128/mBio.01412-17.7 FIG S7  Assessing folding state of EarP_*Ppu*_ variants. To check whether a given variant is properly folded, a ^1^H-^15^N HSQC of ^15^N-labeled variants was recorded (colored) and overlaid with a spectrum of wild-type protein (grey). Note that the mutant spectra were recorded with a small number of scans. Spectra of all variants except the D274A variant overlap well with the wild-type spectrum and show well-dispersed peaks (an indicator of a stable secondary structure), confirming that the single-amino-acid substitutions do not influence proper folding of EarP. In contrast, the D274A variant is in large part unfolded. Download FIG S7, TIF file, 3.1 MB. Copyright © 2017 Krafczyk et al. 2017 Krafczyk et al. This content is distributed under the terms of the Creative Commons Attribution 4.0 International license.

In parallel, a bacterial two-hybrid analysis (44) was set up to investigate interactions between EF-P_*Ppu*_ and WT^EarP^ as well as the above-mentioned nine substitution variants (Fig. 5B). Therefore, fusions were generated with two complementary fragments, T25 and T18, encoding segments of the catalytic domain of the *Bordetella pertussis* adenylate cyclase CyaA. If EF-P_*Ppu*_ and WT^EarP^ do interact, then CyaA is reconstituted, which in turn allows induction of the *lac* promoter and results in *lacZ* expression. Accordingly, β-galactosidase activity is a measure of the interaction strength. When coproducing EF-P_*Ppu*_ with WT^EarP^, we determined ca. 250 MU, whereas combinations with solely T25 and T18 resulted in <60 MU, thus defining the threshold of the assay (Fig. S1C). Except for the R271A^EarP^ and Y291A^EarP^ proteins, all other variants were below this threshold, indicating that alterations in the donor binding site might also affect acceptor binding (Fig. S1C).

### The KOW-like EF-P N-domain is sufficient for EarP-mediated rhamnosylation.

**FIG 6:**
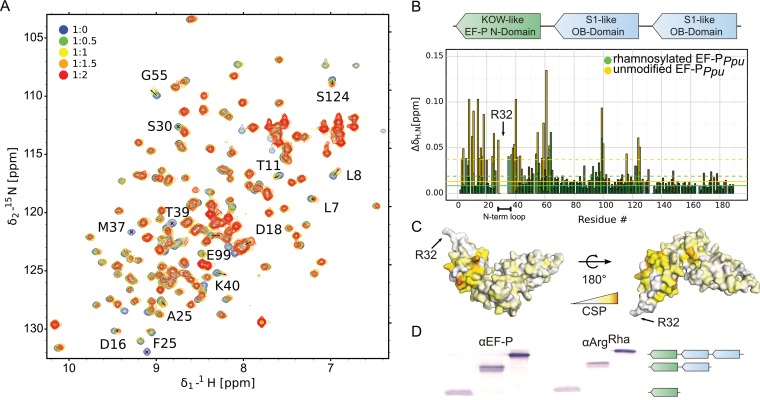
Interaction of EF-P_*Ppu*_ with EarP_*Ppu*_. (A) NMR titration of unmodified EF-P_*Ppu*_ titrated by EarP_*Ppu*_. Overlay of ^1^H-^15^N HSQC spectra of EF-P recorded at different titration steps. EF-P was titrated in a 1:2 EF-P_*Ppu*_/EarP_*Ppu*_ molar ratio. Color coding for respective titration steps is indicated in the upper left corner. Examples of peaks with high chemical-shift perturbations (CSPs) or severe line broadening are shown by labels indicating the assignment of given peaks. (B, top) Domain structure of EF-P. EF-P consists of three β-barrel domains. The KOW-like EF-P N-domain harbors the rhamnosylation target R32^EF-P^. (Bottom) CSPs of EF-P_*Ppu*_ titrated by EarP_*Ppu*_ derived from panel A. Unmodified and rhamnosylated EF-P_*Ppu*_ proteins were titrated by EarP_*Ppu*_ to a 1:2 EF-P_*Ppu*_/EarP_*Ppu*_ molar ratio. To analyze the interaction, CSPs were calculated as described in Materials and Methods and plotted against residue numbers. Color coding is indicated in the upper right corner. Full lines indicate median CSPs, dashed lines indicate median CSPs plus standard deviations, and residues with CSPs higher than the median plus standard deviation are shown in brighter shades of the colors. The N-terminal loop containing rhamnosylation target R32^EF-P^ is indicated. (C) CSPs of unmodified EF-P_*Ppu*_ titrated by EarP_*Ppu*_ plotted on the model of EF-P from *P. aeruginosa* (45) (PDB accession number 3OYY) using a white-to-orange gradient, where white represents the weakest CSP and orange depicts the strongest CSP. The position of R32^EF-P^ is indicated. (D) Rhamnosylation experiments using full-length EF-P_*Ppu*_ and C-terminally truncated variants (EF-P_*Ppu*_ with aa 1 to 128, EF-P_*Ppu*_ with aa 1 to 65). EF-P was detected using 0.2 µg/ml anti-EF-P. Rhamnosylation of purified protein was detected using 0.25 µg/ml anti-Arg^Rha^. The domain structure of the respective protein variants is indicated as in panel B.

To test which part of EF-P is involved in the interaction with EarP, NMR chemical shift perturbation experiments were performed by comparing ^1^H-^15^N HSQC results between unbound EF-P_*Ppu*_ and EarP_*Ppu*_-bound EF-P_*Ppu*_ (Fig. 6A). Triple-resonance experiments of EF-P_*Ppu*_ enabled backbone assignment, with a sequence coverage of 97%. Missing assignments are for residues S123, R133, N140, V164, D175, and G185. The assignment also enabled secondary-structure determination from secondary chemical shifts and confirmed the validity of the EF-P model for *P. putida*, based on the crystal structure of *P. aeruginosa* EF-P (Fig. S3E) (45). The titration experiment showed clear chemical shift perturbations in the N-terminal acceptor domain of EF-P_*Ppu*_ (Fig. 6B and C). However, R32^EF-P^ and residues surrounding the rhamnosylation site (e.g., S30^EF-P^, G31^EF-P^, R32^EF-P^, N33^EF-P^) are severely line broadened beyond detection. Therefore, chemical shift perturbation values cannot be determined for these and vicinal residues. This line broadening is an indication that they are bound by EarP_*Ppu*_ and thus have rotational correlation times expected for a complex of that size. Several residues located in the S1-like OB-domain are also slightly affected. However, this is not necessarily due to direct contacts with EarP_*Ppu*_ but might also be propagating effects. Therefore, we also investigated *in vitro* rhamnosylation of truncated EF-P_*Ppu*_ variants comprising either amino acids 1 to 128 or amino acids 1 to 65 (Fig. 6D). Both truncations were readily rhamnosylated by EarP_*Ppu*_, further corroborating that EF-P contact sites are predominantly located in the KOW-like N-domain.

In addition, we compared NMR interactions between EarP_*Ppu*_ and unmodified EF-P_*Ppu*_ or rhamnosylated EF-P_*Ppu*_. This experiment clearly showed that chemical shift perturbations for unmodified EF-P are stronger than for rhamnosylated EF-P (Fig. 6B). Thus, EarP releases EF-P after rhamnosylation due to decreased affinity, while unmodified EF-P binds with higher affinity to enable efficient posttranslational modification.

### Mutational analysis of the three invariant EarP residues D13, D17, and E273.

**FIG 7:**
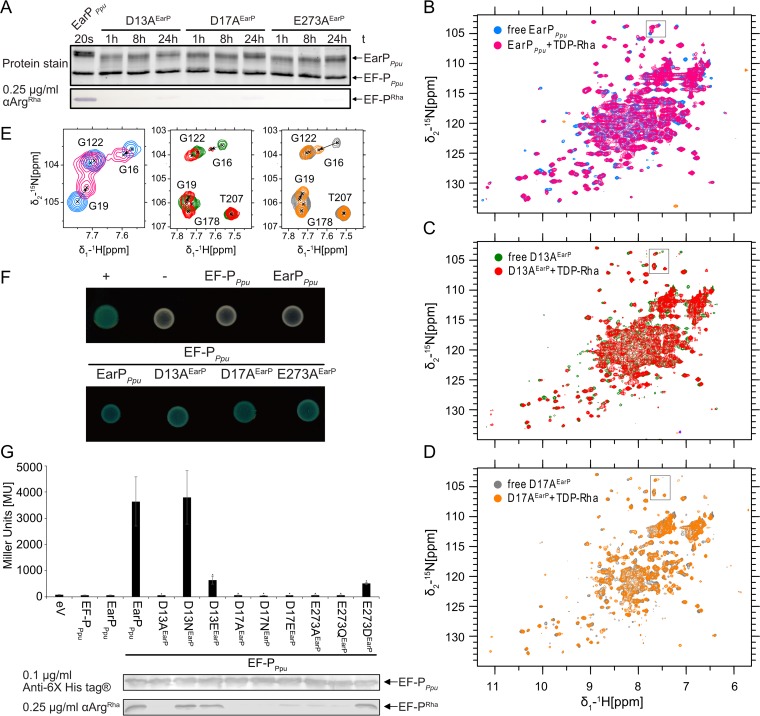
Mutational analysis of the three invariant EarP residues D13, D17, and E273. (A) *In vitro* rhamnosylation of EF-P_*Ppu*_ by single-amino-acid exchange variants, specifically, D13A^EarP^, D17A^EarP^, and E273A^EarP^ variants. EF-P (2.5 µM) and TDP-Rha (1 mM) were incubated together with the EarP_*Ppu*_ variants (0.5 µM) and sampled at different time points. Rhamnosylated EF-P_*Ppu*_ (EF-P^Rha^) was detected after Western blotting using 0.25 µg/ml anti-Arg^Rha^. (B) Overlay of ^1^H-^15^N HSQC spectra of wild-type EarP_*Ppu*_ that was free and bound to TDP-Rha. (C) Overlay of ^1^H-^15^N HSQC spectra of the free and TDP-Rha-bound D13A^EarP^ variant. (D) Overlay of ^1^H-^15^N HSQC spectra of the free and TDP-Rha-bound D17A^EarP^ variant. The color coding is indicated in the upper left corner of each spectrum. The titrations are described in detail in Materials and Methods. (E) Zoom into the overlaid spectra shown in panels B, C, and D. The position of the zoom is indicated by a black frame in the corresponding original overlay. Peak assignments are shown. The movement of G16 and G19 upon TDP-Rha titration is indicated by dashed arrows. (F) Bacterial two-hybrid analysis of protein-protein interactions between EarP_*Ppu*_, the D13A^EarP^, D17A^EarP^, and E273A^EarP^ variants, and the protein acceptor EF-P_*Ppu*_ in *E. coli* BTH101. The blue color of colonies results from cleavage of X-Gal by β-galactosidase and indicates protein-protein interaction between coexpressed hybrids. (G, top) Analysis of *in vivo* activities of EarP_*Ppu*_, D13A^EarP^, D17A^EarP^, and E273A^EarP^. *In vivo* EarP_*Ppu*_ activities were determined by measuring the β-galactosidase activities of an *E. coli* MG1655 P_*cadBA*_::*lacZ* Δ*efp* strain heterologously expressing *efp*_*Ppu*_ together with the wild-type or mutant *earP*_*Ppu*_ genes from o/n cultures in LB (pH 5.8). Means of three independent measurements are shown. Standard deviations from three independent experiments were determined. (Bottom) Western blot analysis of o/n cultures of *E. coli* MG1655 P_*cadBA*_::*lacZ* Δ*efp* heterologously expressing *efp*_*Ppu*_ together with the wild-type or *earP*_*Ppu*_ mutants. Rhamnosylated EF-P_*Ppu*_ (EF-P^Rha^) was detected using 0.25 µg/ml anti-Arg^Rha^.

We and others previously showed that EarP inverts the anomeric configuration on the sugar moiety from TDP-β-l-rhamnose to α-rhamnosyl arginine (26, 27). Reportedly, inverting glycosyltransferases employ a direct-displacement S_N_2-like reaction (46). The molecular basis for inverted N-linked glycosylation was elucidated for the oligosaccharyl transferase PglB (47). Here the catalytic site features three acidic side chains (29). As with PglB, three negatively charged residues—aspartates D13^EarP^ and D17^EarP^ and glutamate E273^EarP^—were identified as potential candidates to catalyze the glycosylation reaction (Fig. 3B). All three residues are invariant in all EarP orthologues (Fig. 4A; Data Set S3). Moreover, the D13^EarP^ and D17^EarP^ variants as well as the E273^EarP^ variant are in the vicinity of the rhamnose moiety and might therefore be proximal to the putative active center and R32 of EF-P (Fig. 3B). The distances of these three residues to rhamnose atoms range from 2.5 to 4.5 Å (the carboxyl group of D13 is the closest, with a distance of 2.5 Å to the methyl group of the rhamnose, followed by the side chains of D17 and E273, with distances of 3.9 and 4.5 Å to the hydroxyl group of C4 and C2, respectively). Consequently, we constructed the corresponding alanine substitution variants D13A^EarP^, D17A^EarP^, and E273A^EarP^ and investigated their enzymatic activities *in vitro*. In line with the idea that these residues might be involved in catalysis, EF-P rhamnosylation could not be detected even after 8 h of incubation, and accordingly these EarP variants are inactive (Fig. 7A).

To exclude misfolding being causative for the nonfunctional EarP_*Ppu*_ protein variants, ^15^N HSQCs were measured for D13A^EarP^, D17A^EarP^, and E273A^EarP^. The spectra show no structural alterations from WT^EarP^ (Fig. 7B, C, and D and see Fig. S7). Additionally, the variants D13A^EarP^ and D17A^EarP^ were titrated with TDP-Rha being indistinguishable from WT^EarP^ perturbations. Interestingly, although D13^EarP^ and D17^EarP^ resonances could not be assigned, other residues in close proximity (G16^EarP^ and G19^EarP^) exhibited strong perturbations not only in WT^EarP^ but also in the D13A^EarP^ and D17A^EarP^ variants upon TDP-Rha binding, despite not forming direct ligand contacts (Fig. 7E). Similarly, we could measure TDP-Rha binding for E273A/D/N^EarP^ variants using STD NMR (Fig. S5C). This confirms that these mutations do not affect donor substrate binding.

To investigate interactions between EF-P_*Ppu*_ and the D13A^EarP^, D17A^EarP^, and E273A^EarP^ variants, we again performed a bacterial two-hybrid analysis and were able to show that all substitution variants are capable of acceptor binding, demonstrated by a blue colony on X-Gal (5-bromo-4-chloro-3-indolyl-β-d-galactopyranoside)-containing LB plates (Fig. 7F, S1C).

To further corroborate our findings on the *in vitro*-inactive D13A^EarP^, D17A^EarP^, and E273A^EarP^ variants, they were subjected to an *in vivo* experiment in which we investigated their ability to activate EF-P_*Ppu*_ (Fig. 5A). Additional substitutions—D13N/E^EarP^, D17N/E^EarP^, and E273Q/D^EarP^—were also included in the study. Expectedly, coproduction of the D13A^EarP^, D17A^EarP^, and E273A^EarP^ variants with EF-P_*Ppu*_ phenocopies Δ*efp* with respect to P_*cadBA*_ activation and *in vivo* rhamnosylation (Fig. 7G; Fig. S1B). Similar results were obtained with the D17N/E^EarP^ and E273Q^EarP^ variants, whereas the D13E^EarP^ and E273D^EarP^ variants were drastically impaired in function, although they retained some residual activity. Their impairment is indicated by a certain degree of P_*cadBA*_ activation as well as a band in the *in vivo* rhamnosylation blot (Fig. 7G; Fig. S1B). In contrast, a variant with a change of D13 to asparagine was indistinguishable from WT^EarP^, implying an importance of the chain length over charge.

Our thorough analysis of these EarP variants suggests that they are promising candidates to be involved in catalysis.

## DISCUSSION

Activation of the proline-specific translation elongation factors EF-P and IF-5A is usually achieved by posttranslational elongation of the ε-amino group of a conserved lysine (20–23, 48, 49). The resultant noncanonical amino acids—β-lysinyl-hydroxylysine, hypusine, and 5-amino-pentanolyl-lysine—appear to be chemically and structurally analogous. We recently showed that in a subset of bacteria, a so-far-unappreciated form of posttranslational modification plays an important role in the activation of EF-P. Here, instead of lysine, the guanidine group of a conserved arginine is modified with a rhamnose moiety by a glycosyltransferase termed EarP (17). This type of modification not only contrasts with the other known EF-P/IF-5A activation strategies but is also one of only two reported cases of enzyme-mediated arginine glycosylation. In canonical N-linked glycosylation, the sugar is attached to the amide nitrogen of an asparagine in an N-X-S/T consensus sequence (X is any amino acid except for a proline) (46, 50). In contrast, the effector glycosyltransferase NleB of enteropathogenic *E. coli N-*acetyl-glucosaminylates (GlcNAc) specifically the arginines at positions 117 and 235 in the death domain-containing proteins FADD and TRADD, respectively (31, 51). This in turn antagonizes the apoptosis of infected cells, thereby blocking a major antimicrobial host response. Notably, EarP shows neither sequential nor structural homologies to the GT-A-type glycosyltransferase NleB, and thus the arginine glycosylation of death domains and EF-P are examples of convergent evolution. Instead EarP seems to be structurally related to MurG. Moreover, and despite the lack of a significant overall sequence similarity, certain residues important for function remain the same. According to these facts, one might speculate that EarP is not simply analogous to MurG but a distinct homologue. Note that MurG is essential for cell wall biosynthesis in both Gram-negative and Gram-positive bacteria, and due to its degree of conservation, it is most likely more ancient then EarP. Although there is no real evidence for this, one might hypothesize about the possibility of a duplication of MurG in a betaproteobacterial progenitor, which is the presumed origin of EarP (17). Subsequently, the sequences of both proteins more and more diverged in consequence of distinct donor and acceptor substrates. This assumption is at least also in line with the theory that NleB (GT-A type) and EarP (GT-B type) are phylogenetically nonrelated enzymes. Accordingly, one can also assume that the molecular mechanisms of the glycosyl transfer reactions in both arginine glycosyltransferases differ. In 2016, Wong Fok Lung and coworkers mutated *nleB* and identified certain residues in NleB either interfering with FADD binding or preventing GlcNAcylation (52). They confirmed the importance of two invariant aspartate residues, D221 and D223, from among the nonfunctional NleB protein variants (31). A catalytic Asp-X-Asp motif is featured by various GT-A glycosyltransferases. Here, the two negatively charged aspartate side chains coordinate a divalent cation that facilitates departure of the nucleoside phosphate. Negatively charged amino acids also play important catalytic roles in inverting GT-B glycosyltransferases (46). In the case of the metal-independent fucosyltransferase FucT (53), for example, the side chain carboxyl groups of D13 and E95 may work as base catalysts (46). Also, the activation of the acceptor amide nitrogen by the lipid donor utilizing bacterial oligosaccharyltransferase PglB depends on the two negatively charged amino acids D56 and E319. These residues abolish the conjugation of the nitrogen electrons and allow the positioning of a free electron pair for the nucleophilic attack onto the anomeric center of the donor substrate (29, 47). Analogously, the invariant negatively charged residues D13^EarP^, D17^EarP^, and E273^EarP^ in the EarP glycosyltransferase family might play a role in activating the R32 guanidino group of EF-P. Especially D17^EarP^ and E273^EarP^—both in close proximity to each other—may form a catalytic dyad (Fig. 3B).

While activation of the acceptor substrate might be driven by the essential amino acids D13^EarP^, D17^EarP^, and E273^EarP^, the nucleotide sugar donor TDP-Rha is bound in a highly conserved cavity of the protein C domain. A cocrystal structure of the putative structural EarP analogue MurG_*Eco*_ with its cognate substrate reveals that aromatic amino acid side chains play important roles in UDP binding (PDB accession number 1NLM) (54). Similar interactions were reported for the protein O-fucosyltransferase POFUT1 (PDB accession number 3ZY6), where F357 is involved in π-stacking with the respective nucleobase (55). Stacking interactions also play a role in EarP, in which the aromatic side chains of F252^EarP^ and F258^EarP^ bind the thymine and ribose moiety of TDP-Rha, respectively. In contrast, contacts with the ribose or the phosphate moieties frequently occur via interactions with side chain amines, hydroxyl groups, and backbone amides (37, 54, 55). Accordingly, this is also the case for EarP.

In GT-B glycosyltransferases, positively charged amino acids are often involved in facilitating leaving group departure. This is achieved by neutralization of evolving negative charges on the phosphate moiety during the glycosyl transfer reaction, as described, e.g., for R261 of MurG_*Eco*_ (PDB accession number 1F0K) (37). Notably, *earP*_*Ppu*_ encodes an invariant R271^EarP^ in the equivalent position and a substitution to alanine (R271A^EarP^) strongly impairs protein function, all of which suggests that they have similar roles in product stabilization.

In GT-B glycosyltransferases, the two Rossmann folds can generally be divided into one donor and one acceptor substrate binding domain (40). As with other glycosyltransferases, the nucleotide sugar is bound by the protein C-domain of EarP. Accordingly, it is worth assuming important binding sites for EF-P in the protein N-domain. Conversely, EF-P presumably contacts EarP by amino acids that are in close proximity to the glycosylation site R32^EF-P^. In agreement with this hypothesis, the EF-P β-lysine ligase EpmA, for example, recognizes EF-P via identity elements in a region located around the *E. coli* EF-P modification site K34 (21, 22, 56). Along the same line, the deoxyhypusine synthase (DHS) can efficiently modify a human eIF-5A fragment comprising only the first 90 amino acids of the protein (57). Similarly, we could show that the KOW-like N-terminal domain of EF-P (Fig. 6B) is sufficient to be glycosylated by EarP (Fig. 6D), being congruent with the NMR titrations of EF-P with EarP (Fig. 6A to C). Upon titration with EarP, the chemical shift perturbations observed were (with a few exceptions) restricted to the first 65 residues.

Taking all of this together, we propose a three-step model for the rhamnosylation of EF-P by its cognate modifier EarP. In the ground state, both the nucleotide sugar binding site in the C-domain and the putative acceptor binding site in the N-domain are unoccupied.

In the donor-bound state, TDP-Rha is coordinated within a highly conserved cavity in the protein C-domain, including an aromatic pocket that surrounds the thymine ring (Fig. 3). Previous studies showed that binding of the donor substrate induces structural alterations in both the N and C-domains of glycosyltransferases (40, 58, 59). In MurG, these rearrangements include rotation of F244, which stacks over the nucleobase to cap the donor binding pocket (37). Notably, in the crystal structure of EarP, a phenylalanine, F252, is in the equivalent position, indicating that this capping interaction is conserved (Fig. 3A) (54).

In the catalytic state, the R32 guanidino group of EF-P might be activated by a mechanism analogous to the one that was reported for the oligosaccharyltransferase PglB (47). Hence, in the EF-P rhamnosylation reaction, R271^EarP^ might stabilize the nucleotide product, thereby facilitating leaving group departure. Upon successful inverting glycosyl transfer from TDP-Rha to R32^EF-P^, presumably by a single S_N_2 displacement reaction, the products are released from the active site of EarP, in turn reverting to the unbound ground state.

We point out that there is most likely no strict sequence of binding events, as NMR measurements demonstrate that EarP can interact with either substrate independently.

Altogether, our structural and biochemical investigation of EarP provides first insights into arginine glycosylation and improves our general understanding of N-linked glycosyl transfer reactions. Additionally, our research might open up new avenues for the development of antimicrobial drugs in order to fight, e.g., *P. aeruginosa* infections.

## MATERIALS AND METHODS

### Bacterial strains and growth conditions.

Strains and plasmids used in this study are listed in Data Set S1 in the supplemental material. *P. putida* and *E. coli* were routinely grown in lysogeny broth (LB) (60, 61) according to the Miller modification (62) at 30°C (for *P. putida*) and 37°C (for *E. coli*), unless indicated otherwise. When required, media were solidified by using 1.5% (wt/vol) agar. If necessary, media were supplemented with 50 µg/ml chloramphenicol, 100 µg/ml kanamycin sulfate, and/or 100 µg/ml ampicillin sodium salt. For promoter induction from P_*BAD*_-containing plasmids (63), l-arabinose was added to a final concentration of 0.2% (wt/vol) in liquid medium. For promoter induction from plasmids comprising the *lac* operator sequences, isopropyl β-d-1-thiogalactopyranoside (IPTG) (Sigma-Aldrich) was added to a final concentration of 1 mM.

### Molecular biology methods.

Enzymes and kits were used according to the manufacturers’ directions. Genomic DNA was obtained according to the protocol of Pospiech and Neumann (64), and plasmid DNA was isolated using a Hi Yield plasmid minikit (Süd-Laborbedarf GmbH). DNA fragments were purified from agarose gels by employing a Hi Yield PCR cleanup and gel extraction kit (Süd-Laborbedarf). Restriction endonucleases were purchased from New England Biolabs (NEB). Sequence amplifications by PCR were performed utilizing the Q5 high-fidelity DNA polymerase (NEB) or the OneTaq DNA polymerase (NEB). Mutations were introduced into the *earP* gene by overlap extension PCR (65, 66). Oligonucleotides used in this study are listed in Data Set S1. All constructs were analyzed by Sanger sequencing (LMU Sequencing Service). Standard methods were performed according to the instructions of Sambrook and Russel (67).

### β-Galactosidase activity assay.

Cells expressing *lacZ* under the control of the *cadBA* promoter were grown in buffered LB (pH 5.8) overnight (o/n) and harvested by centrifugation. β-Galactosidase activities were determined as described in reference 68 in biological triplicates and are given in Miller units (MU) (69). The significance of the results was determined by applying a two-sided Student *t* test and stating a result as significantly different if *P* was <0.05.

### Bacterial two-hybrid analysis.

Protein-protein interactions were detected using the bacterial adenylate cyclase two-hybrid system kit (Euromedex) according to the product manuals. Chemically competent (70) *E. coli* BTH101 cells were cotransformed with pUT18C-*efp*_*Ppu*_ and/or the respective pKT25 variants (pKT25-*earP*, pKT25-D13A, pKT25-D17A, pKT25-F191A, pKT25-Y193A, pKT25-F252A, pKT25-Q255A, pKT25-F258A, pKT25-R271A, pKT25-D274A, pKT25-S275A, pKT25-R278A, pKT25-Y291A, pKT25-E273A) and plated on LB screening medium containing 40 μg/ml 5-bromo-4-chloro-3-indolyl-β-d-galactopyranoside (X-Gal) and 0.5 mM IPTG as well as 50 µg/ml kanamycin sulfate and 100 µg/ml ampicillin sodium salt. Transformants containing pUT18-*zip* and pKT25-*zip* were used as positive controls. Transformants carrying pUT18C and pKT25 vector backbones were used as negative controls. Bacteria expressing interacting protein hybrids exhibit a blue phenotype on screening plates due to functional complementation of the CyaA fragments (T18 and T25). After 48 h of incubation at 30°C, plates containing around 100 colonies were evaluated. Representative colonies were transferred to liquid LB cultures containing kanamycin sulfate and ampicillin sodium salt and incubated o/n at 30°C. Subsequently, 2 µl of the o/n culture were spotted on LB X-Gal–IPTG screening plates. Pictures were taken after 48 h of cultivation at 30°C.

For quantification of interaction strength, which corresponds to the β-galactosidase activity, cells were inoculated in 1.5 ml LB medium containing 0.5 mM IPTG as well as 50 µg/ml kanamycin sulfate and 100 µg/ml ampicillin sodium salt. After incubation in 2-ml reaction tubes under microaerobic conditions at 30°C for 42 h, cells were harvested and β-galactosidase activities were determined as described above.

### Protein purification.

C-terminally His_6_-tagged EarP_*Ppu*_ variants (pBAD33-*earP*_*Ppu*_) were overproduced in *E. coli* LMG194 by addition of 0.2% arabinose to exponentially growing cells and subsequent cultivation at 18°C o/n. N-terminally His_6_-tagged EarP (pACYC-DUET-*earP*_*Ppu*_) and His_6_-SUMO-tagged EF-P_*Ppu*_ (pET-SUMO-*efp*_*Ppu*_) were overproduced in *E. coli* BL21(DE3) by addition of 1 mM IPTG to exponentially growing cells. Subsequently, cells were incubated at 18°C overnight. Rhamnosylated EF-P_*Ppu*_ (EF-P^Rha^) was produced by cooverproduction with His_6_-tagged EarP_*Ppu*_. Cells were lysed by sonication, and His_6_-tagged proteins were purified using Ni-nitrilotriacetic acid (Ni-NTA; Qiagen) according to the manufacturer’s instructions. The His_6_-SUMO tag was removed by incubation with 1 μ/mg His_6_-Ulp1 (71) overnight. Subsequently, tag-free EF-P_*Ppu*_ was collected from the flowthrough after metal chelate affinity chromatography. For biochemical analyses, cells were cultivated in LB. For use in NMR spectroscopy, cells were grown in M9 minimal medium (62). If necessary, ^15^N-labeled nitrogen (^15^NH_4_Cl) and ^13^C-labeled glucose were used. For NMR backbone assignment of EarP_*Ppu*_, additionally 99.8%-pure heavy water D_2_O (Sigma-Aldrich) was used instead of H_2_O in growth medium to allow partial deuteration of the protein in order to reduce cross-relaxation effects and increase the signal-to-noise ratio. Size exclusion chromatography of EarP_*Ppu*_ and the D274A^EarP^ variant was performed in 100 mM NaP_i_ (pH 7.6) 50 mM NaCl using a Superdex 200 Increase 10/300-Gl column with a flow rate of 0.3 ml/min on an Äkta purifier (GE Healthcare). Four milligrams of protein was loaded in a volume of 0.5 ml (8 mg/ml). Eluting protein was detected at 280 nm. Fractions of 0.5 ml were collected.

For the production of selenomethylated EarP_*Ppu*_, *E. coli* BL21(DE3) cells expressing N-terminally His_6_-tagged EarP_*Ppu*_ were cultivated in 1 liter M9 minimal medium at 37°C to an optical density at 600 nm (OD_600_) of 0.6. One hundred micrograms of threonine, 100 µg lysine, and 50 µg isoleucine were added to feedback inhibit methionine biosynthesis (72). Additionally, 50 µg l-(+)-selenomethionine was added 15 min prior to induction. Protein production was induced by addition of 1 mM IPTG, and cells were incubated at 18°C overnight. Protein concentrations were determined as described by Bradford (73). For biochemical analyses, EarP_*Ppu*_ and EF-P_*Ppu*_ were dialyzed against 100 mM NaP_i_, pH 7.6, 5 mM dithiothreitol (DTT), whereas a buffer composed of 100 mM NaP_i_, pH 7.6, 50 mM NaCl, and 5 mM DTT was used when the proteins were subjected to NMR analysis.

### Synthesis of a single rhamnosyl-arginine containing glycopeptide.

**FIG 8:**
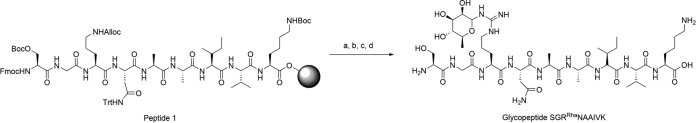
Synthesis of glycopeptide SGR^Rha^NAAIVK. a, SiPhH_3_ (phenylsilane), Pd(PPh_3_)_4_, CH_2_Cl_2_; b, 1-(*tert*-butoxycarbonyl)-3-(2,3,4-tri-*O*-acetyl-6-deoxy-l-mannopyranos-1-yl)-2-ethyl-isothiourea (26) AgNO_3_ (silver nitrate), NEt_3_ (triethylamine), DMF; c, N_2_H_4_·H_2_O (5% solution in DMF); d, TFA-H_2_O-phenol-TIPS (88/5/5/2).

Moisture- and air-sensitive reactions were conducted in flame-dried glassware under an argon atmosphere. Commercially available reagents and solvents were used without further purification. CH_2_Cl_2_ was distilled from calcium hydride, and tetrahydrofuran (THF) was distilled from sodium benzophenone immediately prior to use. Dimethylformamide (DMF) was stored under argon in a flask containing 4 Å molecular sieves. Reactions were monitored by thin layer chromatography (TLC) with precoated Silica Gel 60 F_254_ aluminum plates (Merck, Darmstadt, Germany) using UV light and methoxyphenol reagent (100 ml 0.2% ethanolic methoxyphenol solution and 100 ml 2 M ethanolic sulfuric acid) as the visualizing agent. Flash chromatography was performed using silica gel (35 to 70 μm) from Acros Organics. Peptide purification by reverse-phase high-performance liquid chromatography (RP-HPLC) was performed on a JASCO purification system with a UV–visible-light detector (model UV-2075Plus) using a Phenomenex Aeris Peptide 5-μm XB-C_18_ column (250 by 21.2 mm). Analytical RP-HPLC was measured on a JASCO system with a Phenomenex Aeris Peptide 5-μm XB-C_18_ column (250 by 4.6 mm). In all cases, mixtures of water (eluent A) and acetonitrile (eluent B) were used as eluents; if required, 0.1% formic acid (FA) or 0.1% trifluoroacetic acid (TFA) was added. High-resolution electrospray ionization (HR-ESI) mass spectra were recorded on a Thermo Finnegan LTQ FT mass spectrometer or on a Bruker maxis apparatus equipped with a Waters ACQUITY ultrahigh-performance liquid chromatograph (UPLC) using a Kinetex C_18_ column (2.6 µm, 100 Å) at 40°C (Fig. 8).

Glycopeptide SGR^Rha^NAAIVK was synthesized using a Liberty Blue automated microwave peptide synthesizer, followed by on-resin glycosylation and deprotection (Fig. 8). For construction of peptide 1, 0.1 mmol of preloaded H-Lys(Boc)-2-chlorotrityl resin (loading concentration, 0.78 mmol/g) was applied. Cleavage of the Fmoc-protecting group was achieved with 20% piperidine in DMF (75°C, 35 W, 3 min). Fmoc-protected amino acids (5 eq) were activated for peptide coupling using 5 eq of ethyl (hydroxyimino)cyanoacetate (Oxyma Pure), 0.5 eq of *N*,*N*-diisopropylethylamine (DIPEA), and 5 eq of *N*,*N*′-diisopropylcarbodiimide. All coupling reactions were conducted at 75°C and 28 W for 5 min. Removal of the allyloxycarbonyl-protecting group and subsequent coupling of the sugar moiety, as well as deprotection of the acetyl groups, were performed according to established procedures (26). Final deprotection gave the desired glycopeptide, SGR^Rha^NAAIVK, yielding 39% after HPLC purification. The amino acid sequence of the glycopeptide corresponds to the primary structure of the *S. oneidensis* acceptor loop, which is highly similar to the consensus sequence of EarP-arginine-type EF-Ps (17).

High-resolution mass spectrometry (HRMS) (ESI^+^), calculated for C_44_H_82_N_14_O_16_ [M+2H]^2+^, *m/z* = 531.3011; found, 531.3016.

**FIG 9:**
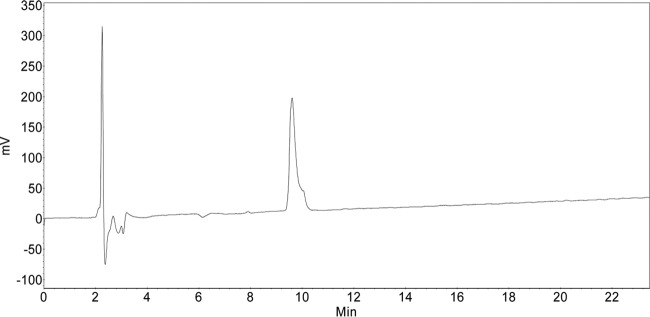
HPLC data.

HPLC (0.1% TFA, 0 min, 8% B → 45 min, 50% B; flow, 1 ml/min), *t*_*R*_ (retention time) = 9.61 min, *λ* = 204 nm (Fig. 9).

### Antibody generation.

Polyclonal antibodies were raised commercially by Eurogentec according to the Rabbit Speedy 28-day (AS superantigen) program. The mono-rhamnosyl-arginine-containing peptide was coupled to bovine serum albumin (BSA) according to an internal protocol (AS-PECO 05). Antibodies capable of binding to rhamnosyl-arginine were purified from rabbit sera by affinity chromatography (AS-PURI−MED) against the glycopeptide SGR^Rha^NAAIVK. To test the specificity of the purified polyclonal antibodies toward EF-P^Rha^, 1.5 µg of unmodified and 0.5 µg of modified EF-P were transferred to a nitrocellulose membrane by Western blotting. While polyclonal antibodies that were raised against EF-P from *S. oneidensis* detect both unmodified and modified EF-P_*Ppu*_, anti-Arg^Rha^ specifically detects the modified protein variant (Fig. S1A).

### SDS-PAGE and Western blotting.

Electrophoretic separation of proteins was carried out using SDS-PAGE as described by Lämmli (74). Separated proteins were visualized in gel using 0.5% (vol/vol) 2-2-2-trichloroethanol (75) and transferred onto a nitrocellulose membrane by vertical Western blotting. Antigens were detected using 0.1 µg/ml anti-His_6_ tag (Abcam, Inc.), 0.2 μg/ml anti-EF-P, or 0.25 μg/ml of anti-Arg^Rha^. Primary antibodies (rabbit) were targeted by 0.2 μg/ml alkaline phosphatase-conjugated anti-rabbit IgG (H&L) (goat) antibody (Rockland). Target proteins were visualized by addition of substrate solution (50 mM sodium carbonate buffer, pH 9.5, 0.01% [wt/vol] nitroblue tetrazolium, 0.045% [wt/vol] 5-bromo-4-chloro-3-indolylphosphate).

### Determination of kinetic parameters.

Kinetic parameters were determined by varying TDP-Rha concentrations while keeping concentrations of EarP_*Ppu*_ (0.1 µM) and unmodified EF-P_*Ppu*_ (2.5 µM) constant. A mixture of EarP_*Ppu*_ and unmodified EF-P_*Ppu*_ was equilibrated to 30°C in 100 mM NaP_i_ (pH 7.6). The reaction was started by the addition of TDP-Rha and was stopped after 20 s of incubation at 30°C by the addition of 1 vol of 2× Lämmli buffer (74) and incubation at 95°C for 5 min. Samples were subjected to SDS-PAGE, and rhamnosylated EF-P_*Ppu*_ was detected as described above. Band intensities were quantified using ImageJ (76). Product formation (in nanomoles per milligram) was calculated relative to fully (*in vivo*) rhamnosylated EF-P_*Ppu*_. *K*_*m*_ and *k*_cat_ values were determined by fitting reaction rates (in nanomoles per milligram per second) to the Michaelis-Menten equation using SigmaPlot. Time course experiments conducted at a TDP-Rha concentration of 500 µM show that the rhamnosylation reaction is not saturated after 20 s of incubation (Fig. S2A).

### Fold recognition.

Fold recognition models were generated using the online user interface of Phyre^2^ (33, 77), SWISS-MODEL (78–81), and the I-TASSER server (34–36) as instructed on the websites. Model structures were selected from the array of results according to best confidence, Q mean, and *z* scores, respectively. All images of tertiary protein structures in this work were generated using the UCSF Chimera package developed by the Resource for Biocomputing, Visualization, and Informatics at the University of California, San Francisco (82). Protein structures were obtained as .pdb files from http://www.rcsb.org (83) or the respective modeling platforms mentioned above.

### Determination of intracellular TDP-Rha concentrations.

Cells were grown in 1 liter LB to an OD_600_ of 0.5 (5 × 10^8^ cells/ml), harvested by centrifugation, and resuspended in 25 ml 100 mM NaP_i_ (pH 7.6) (2 × 10^10^ cells/ml). After disruption of cells with a Constant Systems Ltd. continuous-flow cabinet at 1.35 kb, cell debris were removed by centrifugation and lysates were sterilized by filtration (Steriflip). A mixture of EarP_*Ppu*_ (0.1 µM) and unmodified EF-P_*Ppu*_ (2.5 µM) was equilibrated to 30°C in 10 µl 100 mM NaP_i_ (pH 7.6). The reaction was started by addition of 10 µl lysate from ~2 × 10^7^ or ~2 × 10^8^ cells and stopped after 20 s of incubation at 30°C by addition of 1 vol 2× Lämmli buffer (74) and incubation at 95°C for 5 min. In parallel, a TDP-Rha calibration series was generated by addition of TDP-Rha at final concentrations ranging from 5 µM to 160 µM, including the linear range of the rhamnosylation reaction rate (Fig. 5D). Samples were subjected to SDS-PAGE, and rhamnosylated EF-P_*Ppu*_ was detected as described above. Band intensities were quantified using ImageJ (76). TDP-Rha concentrations in samples containing lysate were calculated by dividing the respective relative band intensities by the slope of the corresponding calibration curve (5 µM to 80 µM TDP-Rha). Intracellular TDP-Rha concentrations were calculated from the amount of substance (in moles) per cell, with an assumption of equal distribution of TDP-Rha across all cells as well as an average cell volume of 3.9 µm^3^ for *E. coli* (84) and 2.1 µm^3^ for *P. putida* and *P. aeruginosa* (85).

### NMR spectroscopy and backbone assignment of EF-P and EarP.

All NMR experiments were performed at 298 K on Bruker Avance III spectrometers with a magnetic field strength corresponding to a proton Larmor frequency of 600 MHz (equipped with a Bruker TXI cryogenic probe head), 700 MHz (equipped with a Bruker room temperature probe head), or 800 MHz (equipped with a Bruker TXI cryogenic probe head). All data sets were processed using NMRPipe (91).

Before NMR measurements of ^15^N- and ^13^C-labeled EF-P (700 µM) in 100 mM NaP_i_, 50 mM NaCl, and 5 mM DTT (pH 7.6), 0.02% NaN_3_ was added to the sample. Sequential resonance assignment was obtained from two-dimensional (2D) 1H-15N HSQC and three-dimensional (3D) HNCA, CBCACONH, and HNCACB backbone experiments, using a constant time during ^13^C evolution (86). The assignment process was assisted by CARA (http://cara.nmr.ch) and CcpNmr Analysis (63), and 98% of the backbone resonances could be assigned. Missing assignments for residues other than prolines are S123, R133, N140, V164, D175, and G185. Secondary chemical shift analysis was performed based on the difference between measured ^13^C_α_ and ^13^C_β_ chemical shifts and random coil chemical shifts of the same nuclei to assign a secondary structure to the EF-P sequence (Fig. S3E) and confirm the validity of the model shown in Fig. 6 (87, 88).

Due to the size of EarP (43 kDa), backbone resonance assignment was possible only for ^2^H-, ^15^N-, and ^13^C-labeled samples to reduce the number of protons and thus cross-relaxation effects, which also enables efficient acquisition of backbone assignment experiments in TROSY mode (89). TROSY-HNCA, -HNCACB, and -CBCACONH experiments (90), processed by NMRPipe (91) and analyzed using CARA (http://cara.nmr.ch), enabled backbone resonance assignment of 62% of all assignable residues (excluding prolines).

The NMR titrations were always performed by adding an unlabeled interaction partner to the ^15^N-labeled protein sample and monitoring the progress of the titration by recording ^1^H-^15^N HSQC. First, ^15^N-labeled 150 µM unmodified EF-P was titrated with unlabeled EarP to a 1:2 EF-P/EarP molar ratio with intermediate steps at 1:0, 1:0.5, 1:1, and 1:1.5 EF-P/EarP molar ratios. ^15^N-labeled 41 µM rhamnosylated EF-P was titrated with unlabeled EarP to a 1:2 EF-P/EarP molar ratio without any intermediate steps. ^15^N-labeled 540 µM wild-type EarP was titrated with unlabeled TDP-Rha to a 1:5 EarP/TDP-Rha molar ratio with intermediate steps at 1:0, 1:0.2, 1:1, and 1:3 molar ratios. ^15^N-labeled 186 µM D13A variant or 209 µM D17A EarP variant was titrated by the addition of TDP-Rha to an approximately 1:10 molar ratio with no intermediate steps. To analyze the EF-P/EarP and wild-type EarP/TDP-Rha ratio titration, the chemical-shift perturbations (CSPs) were calculated according to the formula $\text{CSPs}=\sqrt{\Delta \sigma_{H}^{2}+{\left(\Delta \sigma_{N}\times 0.15\right)}^{2}}$, where 0.15 is the weighting factor to account for nitrogen resonances generally spanning a broad frequency range.

To check proper folding of EarP variants, ^1^H-^15^N HSQC spectra of ^15^N-labeled EarP variants with the following single-amino-acid substitutions at the indicated concentrations were recorded: 209 µM D13A, 209 µM D17A, 162 µM F191A, 197 µM Y193A, 139 µM D274A, 186 µM R271A, and 162 µM Y291A.

STD NMR experiments were performed with 10 µM WT^EarP^ or mutants and either 70 µM (1:7 ratio of protein to ligand to mimic SAXS conditions) or 1 mM TDP-Rha in 100 mM potassium phosphate buffer, pH 7.5, 150 mM NaCl, 1 mM DTT, and 10% D_2_O. The experiments were performed on a Bruker Avance III 700-MHz spectrometer equipped with a triple resonance (TXI) room temperature probe head at 277 K. Protein was saturated with 49-ms Gaussian pulses at the resonance frequency of methyl resonances at 0.592 ppm. The experimental results were collected after a total saturation time of 20 s, with 1,596 scans performed for the WT^EarP^ sample with a 100-fold excess of ligand, and after a total saturation time of 5 s, with 4,096 scans performed for the WT^EarP^ sample with a 7-fold excess of ligand. For EarP mutants, the experimental results were collected after a total saturation time of 4 s and with 128 scans.

### Small-angle X-ray scattering.

Thirty microliters of EarP, EarP plus TDP-rhamnose, and buffer (with and without TDP-rhamnose) were measured at 20°C at BioSAXS beamline BM29 at the European Synchrotron Radiation Facility using a 2D Pilatus detector. For each measurement, 10 frames with a 1-s exposure time per frame were recorded for each EarP and buffer sample, using an X-ray wavelength (λ) of 0.9919 Å. Measurements were performed in flow mode, where samples are pushed through a capillary at a constant flow rate to minimize radiation damage. The protein concentrations measured were 1.0, 2.0, 4.0, and 8.0 mg/ml. TDP-Rha was used in a 7:1 excess (ligand to protein). The buffer measurements were subtracted from each protein sample, and the low Q range of 1.0 mg/ml was merged with the high Q range of the 8.0-mg/ml sample, using PRIMUS (92). The merging was done due to the rising scattering density at low Q ranges for the more highly concentrated samples, indicative of aggregation. CRYSOL (93) was used to fit the back-calculated scattering densities from the crystal structure to the experimental data.

### X-ray crystallography.

For crystallization, N-terminally His_6_-tagged EarP_*Ppu*_ expressed as a seleno-methionine derivative was used. The protein was dialyzed to 50 mM Tris, 100 mM NaCl, 1 mM DTT, pH 7.6, and concentrated to 183 µM. TDP-Rha was added to a final concentration of 10 mM. The crystallization condition was 0.2 M ammonium acetate, 0.1 M bis-Tris (pH 6.0), and 27% (wt/vol) polyethylene glycol 3350. A full data set was collected at the ID29 beamline, ESRF, Grenoble, France, at a wavelength of 0.97 Å (the absorption peak for selenium) and with a 15.05% beam transmission with a 0.15° oscillation range, 0.037-s exposure time, and 2,400 frames. The space group was determined to be I4. The data set was phased using single-wavelength anomalous diffraction (SAD) by the Crank2 (94) automatic pipeline in CCP4 (95), using Afro provided by N. S. Pannu (unpublished) for substructure factor amplitude (FA) estimation, Crunch2 (96) for substructure detection, and Solomon (97) for density modification. The anomalous signal extended to a 3.4-Å resolution (in a data set with a 3-Å resolution). We could successfully find 3 Se-Met signals with an occupancy of 1 located in the C-terminal domain and 2 Se-Met signals with an occupancy of ~0.5 located in the N-terminal domain. The initial structure was built in Phenix Autobuild (98), completed with several rounds of manual model building in Coot (99), and used as the model for molecular replacement (MR) of a native data set extending to 2.3 Å. Despite our rigorous efforts in manual model building, which included extreme density modification, use of homology models to model the N-terminal domain, Rosetta modeling, and refinement strategies with different refinement software (Phenix [98], refmac [100], and CNS [101, 102] [and CNS-DEN-assisted refinement]), the structure displays an R-free of 35% at 2.3 Å, with large parts of the electron density in the N-domain not interpretable. No crystallographic pathology (twinning, anisotropy) could be identified in any of the multiple data sets that we obtained, and trying to interpret crystallographic symmetry as noncrystallographic symmetry by deliberately choosing space groups with lower symmetry (C2, P1) did not improve the density. This indicates intrinsic crystal disorder caused by the N-terminal domain adopting several conformations in different unit cells.

### Accession number(s).

Atomic coordinates and structure factors for the reported crystal structures have been deposited with the Protein Data Bank under accession number 5NV8.

## References

[1] Varenne S, Buc J, Lloubes R, Lazdunski C 1984 Translation is a non-uniform process. Effect of tRNA availability on the rate of elongation of nascent polypeptide chains. J Mol Biol 180:549–576. doi:10.1016/0022-2836(84)90027-5. 6084718

[2] Pavlov MY, Watts RE, Tan Z, Cornish VW, Ehrenberg M, Forster AC 2009 Slow peptide bond formation by proline and other N-alkylamino acids in translation. Proc Natl Acad Sci U S A 106:50–54. doi:10.1073/pnas.0809211106. 19104062 PMC2629218

[3] Tanner DR, Cariello DA, Woolstenhulme CJ, Broadbent MA, Buskirk AR 2009 Genetic identification of nascent peptides that induce ribosome stalling. J Biol Chem 284:34809–34818. doi:10.1074/jbc.M109.039040. 19840930 PMC2787343

[4] Woolstenhulme CJ, Parajuli S, Healey DW, Valverde DP, Petersen EN, Starosta AL, Guydosh NR, Johnson WE, Wilson DN, Buskirk AR 2013 Nascent peptides that block protein synthesis in bacteria. Proc Natl Acad Sci U S A 110:E878–E887. doi:10.1073/pnas.1219536110. 23431150 PMC3593848

[5] Gutierrez E, Shin BS, Woolstenhulme CJ, Kim JR, Saini P, Buskirk AR, Dever TE 2013 eIF5A promotes translation of polyproline motifs. Mol Cell 51:35–45. doi:10.1016/j.molcel.2013.04.021. 23727016 PMC3744875

[6] Pelechano V, Alepuz P 2017 eIF5A facilitates translation termination globally and promotes the elongation of many non polyproline-specific tripeptide sequences. Nucleic Acids Res doi:10.1093/nar/gkx479. PMC5499558 28549188

[7] Schuller AP, Wu CC, Dever TE, Buskirk AR, Green R 2017 eIF5A functions globally in translation elongation and termination. Mol Cell 66:194–205.e5. doi:10.1016/j.molcel.2017.03.003. 28392174 PMC5414311

[8] Doerfel LK, Wohlgemuth I, Kothe C, Peske F, Urlaub H, Rodnina MV 2013 EF-P is essential for rapid synthesis of proteins containing consecutive proline residues. Science 339:85–88. doi:10.1126/science.1229017. 23239624

[9] Hersch SJ, Wang M, Zou SB, Moon KM, Foster LJ, Ibba M, Navarre WW 2013 Divergent protein motifs direct elongation factor P-mediated translational regulation in *Salmonella enterica* and *Escherichia coli*. mBio 4:e00180-13. doi:10.1128/mBio.00180-13. 23611909 PMC3638311

[10] Peil L, Starosta AL, Lassak J, Atkinson GC, Virumäe K, Spitzer M, Tenson T, Jung K, Remme J, Wilson DN 2013 Distinct XPPX sequence motifs induce ribosome stalling, which is rescued by the translation elongation factor EF-P. Proc Natl Acad Sci U S A 110:15265–15270. doi:10.1073/pnas.1310642110. 24003132 PMC3780873

[11] Ude S, Lassak J, Starosta AL, Kraxenberger T, Wilson DN, Jung K 2013 Translation elongation factor EF-P alleviates ribosome stalling at polyproline stretches. Science 339:82–85. doi:10.1126/science.1228985. 23239623

[12] Elgamal S, Katz A, Hersch SJ, Newsom D, White P, Navarre WW, Ibba M 2014 EF-P dependent pauses integrate proximal and distal signals during translation. PLoS Genet 10:e1004553. doi:10.1371/journal.pgen.1004553. 25144653 PMC4140641

[13] Starosta AL, Lassak J, Peil L, Atkinson GC, Virumäe K, Tenson T, Remme J, Jung K, Wilson DN 2014 Translational stalling at polyproline stretches is modulated by the sequence context upstream of the stall site. Nucleic Acids Res 42:10711–10719. doi:10.1093/nar/gku768. 25143529 PMC4176338

[14] Woolstenhulme CJ, Guydosh NR, Green R, Buskirk AR 2015 High-precision analysis of translational pausing by ribosome profiling in bacteria lacking EFP. Cell Rep 11:13–21. doi:10.1016/j.celrep.2015.03.014. 25843707 PMC4835038

[15] Hanawa-Suetsugu K, Sekine S, Sakai H, Hori-Takemoto C, Terada T, Unzai S, Tame JR, Kuramitsu S, Shirouzu M, Yokoyama S 2004 Crystal structure of elongation factor P from *Thermus thermophilus* HB8. Proc Natl Acad Sci U S A 101:9595–9600. doi:10.1073/pnas.0308667101. 15210970 PMC470720

[16] Blaha G, Stanley RE, Steitz TA 2009 Formation of the first peptide bond: the structure of EF-P bound to the 70S ribosome. Science 325:966–970. doi:10.1126/science.1175800. 19696344 PMC3296453

[17] Lassak J, Keilhauer EC, Fürst M, Wuichet K, Gödeke J, Starosta AL, Chen JM, Søgaard-Andersen L, Rohr J, Wilson DN, Häussler S, Mann M, Jung K 2015 Arginine-rhamnosylation as new strategy to activate translation elongation factor P. Nat Chem Biol 11:266–270. doi:10.1038/nchembio.1751. 25686373 PMC4451828

[18] Doerfel LK, Wohlgemuth I, Kubyshkin V, Starosta AL, Wilson DN, Budisa N, Rodnina MV 2015 Entropic contribution of elongation factor P to proline positioning at the catalytic center of the ribosome. J Am Chem Soc 137:12997–13006. doi:10.1021/jacs.5b07427. 26384033

[19] Lassak J, Wilson DN, Jung K 2016 Stall no more at polyproline stretches with the translation elongation factors EF-P and IF-5A. Mol Microbiol 99:219–235. doi:10.1111/mmi.13233. 26416626

[20] Bailly M, de Crécy-Lagard V 2010 Predicting the pathway involved in post-translational modification of elongation factor P in a subset of bacterial species. Biol Direct 5:3. doi:10.1186/1745-6150-5-3. 20070887 PMC2821294

[21] Navarre WW, Zou SB, Roy H, Xie JL, Savchenko A, Singer A, Edvokimova E, Prost LR, Kumar R, Ibba M, Fang FC 2010 PoxA, YjeK, and elongation factor P coordinately modulate virulence and drug resistance in *Salmonella enterica*. Mol Cell 39:209–221. doi:10.1016/j.molcel.2010.06.021. 20670890 PMC2913146

[22] Yanagisawa T, Sumida T, Ishii R, Takemoto C, Yokoyama S 2010 A paralog of lysyl-tRNA synthetase aminoacylates a conserved lysine residue in translation elongation factor P. Nat Struct Mol Biol 17:1136–1143. doi:10.1038/nsmb.1889. 20729861

[23] Peil L, Starosta AL, Virumäe K, Atkinson GC, Tenson T, Remme J, Wilson DN 2012 Lys34 of translation elongation factor EF-P is hydroxylated by YfcM. Nat Chem Biol 8:695–697. doi:10.1038/nchembio.1001. 22706199

[24] Rajkovic A, Erickson S, Witzky A, Branson OE, Seo J, Gafken PR, Frietas MA, Whitelegge JP, Faull KF, Navarre W, Darwin AJ, Ibba M 2015 Cyclic rhamnosylated elongation factor P establishes antibiotic resistance in *Pseudomonas aeruginosa*. mBio 6:e00823. doi:10.1128/mBio.00823-15. 26060278 PMC4471567

[25] Yanagisawa T, Takahashi H, Suzuki T, Masuda A, Dohmae N, Yokoyama S 2016 *Neisseria meningitidis* translation elongation factor P and its active-site arginine residue are essential for cell viability. PLoS One 11:e0147907. doi:10.1371/journal.pone.0147907. 26840407 PMC4739656

[26] Li X, Krafczyk R, Macošek J, Li YL, Zou Y, Simon B, Pan X, Wu QY, Yan F, Li S, Hennig J, Jung K, Lassak J, Hu HG 2016 Resolving the α-glycosidic linkage of arginine-rhamnosylated translation elongation factor P triggers generation of the first Arg Rha specific antibody. Chem Sci 7:6995–7001. doi:10.1039/c6sc02889f. 28451135 PMC5363779

[27] Wang S, Corcilius L, Sharp PP, Rajkovic A, Ibba M, Parker BL, Payne RJ 2017 Synthesis of rhamnosylated arginine glycopeptides and determination of the glycosidic linkage in bacterial elongation factor P. Chem Sci 8:2296–2302. doi:10.1039/C6SC03847F. 28451332 PMC5363394

[28] Coutinho PM, Deleury E, Davies GJ, Henrissat B 2003 An evolving hierarchical family classification for glycosyltransferases. J Mol Biol 328:307–317. doi:10.1016/S0022-2836(03)00307-3. 12691742

[29] Breton C, Fournel-Gigleux S, Palcic MM 2012 Recent structures, evolution and mechanisms of glycosyltransferases. Curr Opin Struct Biol 22:540–549. doi:10.1016/j.sbi.2012.06.007. 22819665

[30] Singh DG, Lomako J, Lomako WM, Whelan WJ, Meyer HE, Serwe M, Metzger JW 1995 β-Glucosylarginine: a new glucose-protein bond in a self-glucosylating protein from sweet corn. FEBS Lett 376:61–64. doi:10.1016/0014-5793(95)01247-6. 8521968

[31] Pearson JS, Giogha C, Ong SY, Kennedy CL, Kelly M, Robinson KS, Lung TW, Mansell A, Riedmaier P, Oates CV, Zaid A, Mühlen S, Crepin VF, Marches O, Ang CS, Williamson NA, O’Reilly LA, Bankovacki A, Nachbur U, Infusini G, Webb AI, Silke J, Strasser A, Frankel G, Hartland EL 2013 A type III effector antagonizes death receptor signalling during bacterial gut infection. Nature 501:247–251. doi:10.1038/nature12524. 24025841 PMC3836246

[32] Guex N, Peitsch MC 1997 SWISS-MODEL and the Swiss-Pdb Viewer: an environment for comparative protein modeling. Electrophoresis 18:2714–2723. doi:10.1002/elps.1150181505. 9504803

[33] Kelley LA, Sternberg MJ 2009 Protein structure prediction on the Web: a case study using the Phyre server. Nat Protoc 4:363–371. doi:10.1038/nprot.2009.2. 19247286

[34] Zhang Y 2008 I-TASSER server for protein 3D structure prediction. BMC Bioinformatics 9:40. doi:10.1186/1471-2105-9-40. 18215316 PMC2245901

[35] Roy A, Kucukural A, Zhang Y 2010 I-TASSER: a unified platform for automated protein structure and function prediction. Nat Protoc 5:725–738. doi:10.1038/nprot.2010.5. 20360767 PMC2849174

[36] Yang J, Yan R, Roy A, Xu D, Poisson J, Zhang Y 2015 The I-TASSER suite: protein structure and function prediction. Nat Methods 12:7–8. doi:10.1038/nmeth.3213. 25549265 PMC4428668

[37] Ha S, Walker D, Shi Y, Walker S 2000 The 1.9 A crystal structure of *Escherichia coli* MurG, a membrane-associated glycosyltransferase involved in peptidoglycan biosynthesis. Protein Sci 9:1045–1052. doi:10.1110/ps.9.6.1045. 10892798 PMC2144650

[38] Martinez-Fleites C, Macauley MS, He Y, Shen DL, Vocadlo DJ, Davies GJ 2008 Structure of an O-GlcNAc transferase homolog provides insight into intracellular glycosylation. Nat Struct Mol Biol 15:764–765. doi:10.1038/nsmb.1443. 18536723

[39] Lombard V, Golaconda Ramulu H, Drula E, Coutinho PM, Henrissat B 2014 The carbohydrate-active enzymes database (CAZy) in 2013. Nucleic Acids Res 42:D490–D495. doi:10.1093/nar/gkt1178. 24270786 PMC3965031

[40] Liang DM, Liu JH, Wu H, Wang BB, Zhu HJ, Qiao JJ 2015 Glycosyltransferases: mechanisms and applications in natural product development. Chem Soc Rev 44:8350–8374. doi:10.1039/c5cs00600g. 26330279

[41] Viegas A, Manso J, Nobrega FL, Cabrita EJ 2011 Saturation-transfer difference (STD) NMR: a simple and fast method for ligand screening and characterization of protein binding. J Chem Educ 88:990–994. doi:10.1021/ed101169t.

[42] Sievers F, Higgins DG 2014 Clustal Omega, accurate alignment of very large numbers of sequences. Methods Mol Biol 1079:105–116. doi:10.1007/978-1-62703-646-7_6. 24170397

[43] Boels IC, Beerthuyzen MM, Kosters MH, Van Kaauwen MP, Kleerebezem M, De Vos WM 2004 Identification and functional characterization of the *Lactococcus lactis rfb* operon, required for dTDP-rhamnose biosynthesis. J Bacteriol 186:1239–1248. doi:10.1128/JB.186.5.1239-1248.2004. 14973085 PMC344400

[44] Karimova G, Pidoux J, Ullmann A, Ladant D 1998 A bacterial two-hybrid system based on a reconstituted signal transduction pathway. Proc Natl Acad Sci U S A 95:5752–5756. doi:10.1073/pnas.95.10.5752. 9576956 PMC20451

[45] Choi S, Choe J 2011 Crystal structure of elongation factor P from *Pseudomonas aeruginosa* at 1.75 A resolution. Proteins 79:1688–1693. doi:10.1002/prot.22992. 21365687

[46] Lairson LL, Henrissat B, Davies GJ, Withers SG 2008 Glycosyltransferases: structures, functions, and mechanisms. Annu Rev Biochem 77:521–555. doi:10.1146/annurev.biochem.76.061005.092322. 18518825

[47] Lizak C, Gerber S, Numao S, Aebi M, Locher KP 2011 X-ray structure of a bacterial oligosaccharyltransferase. Nature 474:350–355. doi:10.1038/nature10151. 21677752

[48] Park MH, Cooper HL, Folk JE 1982 The biosynthesis of protein-bound hypusine (N^ε^-(4-amino-2-hydroxybutyl)lysine). Lysine as the amino acid precursor and the intermediate role of deoxyhypusine (N^ε^-(4-aminobutyl)lysine). J Biol Chem 257:7217–7222. 6806267

[49] Rajkovic A, Hummels KR, Witzky A, Erickson S, Gafken PR, Whitelegge JP, Faull KF, Kearns DB, Ibba M 2016 Translation control of swarming proficiency in *Bacillus subtilis* by 5-amino-pentanolylated elongation factor P. J Biol Chem 291:10976–10985. doi:10.1074/jbc.M115.712091. 27002156 PMC4900249

[50] Spiro RG 2002 Protein glycosylation: nature, distribution, enzymatic formation, and disease implications of glycopeptide bonds. Glycobiology 12:43R–56R. doi:10.1093/glycob/12.4.43R. 12042244

[51] Li S, Zhang L, Yao Q, Li L, Dong N, Rong J, Gao W, Ding X, Sun L, Chen X, Chen S, Shao F 2013 Pathogen blocks host death receptor signalling by arginine GlcNAcylation of death domains. Nature 501:242–246. doi:10.1038/nature12436. 23955153

[52] Wong Fok Lung T, Giogha C, Creuzburg K, Ong SY, Pollock GL, Zhang Y, Fung KY, Pearson JS, Hartland EL 2016 Mutagenesis and functional analysis of the bacterial arginine glycosyltransferase effector NleB1 from enteropathogenic *Escherichia coli*. Infect Immun 84:1346–1360. doi:10.1128/IAI.01523-15. 26883593 PMC4862703

[53] Sun HY, Lin SW, Ko TP, Pan JF, Liu CL, Lin CN, Wang AH, Lin CH 2007 Structure and mechanism of *Helicobacter pylori* fucosyltransferase. A basis for lipopolysaccharide variation and inhibitor design. J Biol Chem 282:9973–9982. doi:10.1074/jbc.M610285200. 17251184

[54] Hu Y, Chen L, Ha S, Gross B, Falcone B, Walker D, Mokhtarzadeh M, Walker S 2003 Crystal structure of the MurG:UDP-GlcNAc complex reveals common structural principles of a superfamily of glycosyltransferases. Proc Natl Acad Sci U S A 100:845–849. doi:10.1073/pnas.0235749100. 12538870 PMC298689

[55] Lira-Navarrete E, Valero-González J, Villanueva R, Martínez-Júlvez M, Tejero T, Merino P, Panjikar S, Hurtado-Guerrero R 2011 Structural insights into the mechanism of protein O-fucosylation. PLoS One 6:e25365. doi:10.1371/journal.pone.0025365. 21966509 PMC3180450

[56] Katz A, Solden L, Zou SB, Navarre WW, Ibba M 2014 Molecular evolution of protein-RNA mimicry as a mechanism for translational control. Nucleic Acids Res 42:3261–3271. doi:10.1093/nar/gkt1296. 24335280 PMC3950694

[57] Joe YA, Park MH 1994 Structural features of the eIF-5A precursor required for posttranslational synthesis of deoxyhypusine. J Biol Chem 269:25916–25921. 7929297

[58] Qasba PK, Ramakrishnan B, Boeggeman E 2005 Substrate-induced conformational changes in glycosyltransferases. Trends Biochem Sci 30:53–62. doi:10.1016/j.tibs.2004.11.005. 15653326

[59] Ni L, Sun M, Yu H, Chokhawala H, Chen X, Fisher AJ 2006 Cytidine 5'-monophosphate (CMP)-induced structural changes in a multifunctional sialyltransferase from *Pasteurella multocida*. Biochemistry 45:2139–2148. doi:10.1021/bi0524013. 16475803

[60] Bertani G 1951 Studies on lysogenesis. I. The mode of phage liberation by lysogenic *Escherichia coli*. J Bacteriol 62:293–300. 14888646 10.1128/jb.62.3.293-300.1951 PMC386127

[61] Bertani G 2004 Lysogeny at mid-twentieth century: P1, P2, and other experimental systems. J Bacteriol 186:595–600. doi:10.1128/JB.186.3.595-600.2004. 14729683 PMC321500

[62] Miller JH 1972 Experiments in molecular genetics. Cold Spring Harbor Laboratory Press, Cold Spring Harbor, NY.

[63] Guzman LM, Belin D, Carson MJ, Beckwith J 1995 Tight regulation, modulation, and high-level expression by vectors containing the arabinose P_*BAD*_ promoter. J Bacteriol 177:4121–4130. doi:10.1128/jb.177.14.4121-4130.1995. 7608087 PMC177145

[64] Pospiech A, Neumann B 1995 A versatile quick-prep of genomic DNA from gram-positive bacteria. Trends Genet 11:217–218. doi:10.1016/S0168-9525(00)89052-6. 7638902

[65] Ho SN, Hunt HD, Horton RM, Pullen JK, Pease LR 1989 Site-directed mutagenesis by overlap extension using the polymerase chain reaction. Gene 77:51–59. doi:10.1016/0378-1119(89)90358-2. 2744487

[66] Lassak J, Henche AL, Binnenkade L, Thormann KM 2010 ArcS, the cognate sensor kinase in an atypical Arc system of *Shewanella oneidensis* MR-1. Appl Environ Microbiol 76:3263–3274. doi:10.1128/AEM.00512-10. 20348304 PMC2869118

[67] Sambrook J, Russell DW 2001 Molecular cloning. A laboratory manual, 3rd ed. Cold Spring Harbor Laboratory Press, Cold Spring Harbor, NY.

[68] Tetsch L, Koller C, Haneburger I, Jung K 2008 The membrane-integrated transcriptional activator CadC of *Escherichia coli* senses lysine indirectly via the interaction with the lysine permease LysP. Mol Microbiol 67:570–583. doi:10.1111/j.1365-2958.2007.06070.x. 18086202

[69] Miller JH 1992 A short course in bacterial genetics: a laboratory manual and handbook for *Escherichia coli* and related bacteria. Cold Spring Harbor Laboratory, Cold Spring Harbor, NY.

[70] Inoue H, Nojima H, Okayama H 1990 High efficiency transformation of *Escherichia coli* with plasmids. Gene 96:23–28. doi:10.1016/0378-1119(90)90336-P. 2265755

[71] Starosta AL, Lassak J, Peil L, Atkinson GC, Woolstenhulme CJ, Virumäe K, Buskirk A, Tenson T, Remme J, Jung K, Wilson DN 2014 A conserved proline triplet in Val-tRNA synthetase and the origin of elongation factor P. Cell Rep 9:476–483. doi:10.1016/j.celrep.2014.09.008. 25310979 PMC4847715

[72] Galili G 1995 Regulation of lysine and threonine synthesis. Plant Cell 7:899–906. doi:10.1105/tpc.7.7.899. 12242392 PMC160885

[73] Bradford MM 1976 A rapid and sensitive method for the quantitation of microgram quantities of protein utilizing the principle of protein-dye binding. Anal Biochem 72:248–254. doi:10.1016/0003-2697(76)90527-3. 942051

[74] Laemmli UK 1970 Cleavage of structural proteins during the assembly of the head of bacteriophage T4. Nature 227:680–685. doi:10.1038/227680a0. 5432063

[75] Ladner CL, Yang J, Turner RJ, Edwards RA 2004 Visible fluorescent detection of proteins in polyacrylamide gels without staining. Anal Biochem 326:13–20. doi:10.1016/j.ab.2003.10.047. 14769330

[76] Schneider CA, Rasband WS, Eliceiri KW 2012 NIH Image to ImageJ: 25 years of image analysis. Nat Methods 9:671–675. doi:10.1038/nmeth.2089. 22930834 PMC5554542

[77] Kelley LA, Mezulis S, Yates CM, Wass MN, Sternberg MJE 2015 The Phyre2 web portal for protein modeling, prediction and analysis. Nat Protoc 10:845–858. doi:10.1038/nprot.2015.053. 25950237 PMC5298202

[78] Arnold K, Bordoli L, Kopp J, Schwede T 2006 The SWISS-MODEL workspace: a web-based environment for protein structure homology modelling. Bioinformatics 22:195–201. doi:10.1093/bioinformatics/bti770. 16301204

[79] Kiefer F, Arnold K, Künzli M, Bordoli L, Schwede T 2009 The SWISS-MODEL Repository and associated resources. Nucleic Acids Res 37:D387–D392. doi:10.1093/nar/gkn750. 18931379 PMC2686475

[80] Guex N, Peitsch MC, Schwede T 2009 Automated comparative protein structure modeling with SWISS-MODEL and Swiss-Pdb Viewer: a historical perspective. Electrophoresis 30(Suppl 1):S162–S173. doi:10.1002/elps.200900140. 19517507

[81] Biasini M, Bienert S, Waterhouse A, Arnold K, Studer G, Schmidt T, Kiefer F, Gallo Cassarino T, Bertoni M, Bordoli L, Schwede T 2014 SWISS-MODEL: modelling protein tertiary and quaternary structure using evolutionary information. Nucleic Acids Res 42:W252–W258. doi:10.1093/nar/gku340. 24782522 PMC4086089

[82] Pettersen EF, Goddard TD, Huang CC, Couch GS, Greenblatt DM, Meng EC, Ferrin TE 2004 UCSF Chimera—a visualization system for exploratory research and analysis. J Comput Chem 25:1605–1612. doi:10.1002/jcc.20084. 15264254

[83] Berman HM, Westbrook J, Feng Z, Gilliland G, Bhat TN, Weissig H, Shindyalov IN, Bourne PE 2000 The Protein Data Bank. Nucleic Acids Res 28:235–242. doi:10.1093/nar/28.1.235. 10592235 PMC102472

[84] Volkmer B, Heinemann M 2011 Condition-dependent cell volume and concentration of *Escherichia coli* to facilitate data conversion for systems biology modeling. PLoS One 6:e23126. doi:10.1371/journal.pone.0023126. 21829590 PMC3146540

[85] Cohen D, Mechold U, Nevenzal H, Yarmiyhu Y, Randall TE, Bay DC, Rich JD, Parsek MR, Kaever V, Harrison JJ, Banin E 2015 Oligoribonuclease is a central feature of cyclic diguanylate signaling in *Pseudomonas aeruginosa*. Proc Natl Acad Sci U S A 112:11359–11364. doi:10.1073/pnas.1421450112. 26305928 PMC4568660

[86] Sattler M, Schleucher J, Griesinger C 1999 Heteronuclear multidimensional NMR experiments for the structure determination of proteins in solution employing pulsed field gradients. Prog Nucl Magn Reson Spectrosc 34:93–158. doi:10.1016/S0079-6565(98)00025-9.

[87] Schwarzinger S, Kroon GJ, Foss TR, Chung J, Wright PE, Dyson HJ 2001 Sequence-dependent correction of random coil NMR chemical shifts. J Am Chem Soc 123:2970–2978. doi:10.1021/ja003760i. 11457007

[88] Wishart DS, Bigam CG, Holm A, Hodges RS, Sykes BD 1995 13C and 15N random coil NMR chemical shifts of the common amino acids. I. Investigations of nearest-neighbor effects. J Biomol NMR 5:67–81. 7881273 10.1007/BF00227471

[89] Pervushin K, Riek R, Wider G, Wüthrich K 1997 Attenuated T2 relaxation by mutual cancellation of dipole-dipole coupling and chemical shift anisotropy indicates an avenue to NMR structures of very large biological macromolecules in solution. Proc Natl Acad Sci U S A 94:12366–12371. doi:10.1073/pnas.94.23.12366. 9356455 PMC24947

[90] Salzmann M, Pervushin K, Wider G, Senn H, Wüthrich K 1998 TROSY in triple-resonance experiments: new perspectives for sequential NMR assignment of large proteins. Proc Natl Acad Sci U S A 95:13585–13590. doi:10.1073/pnas.95.23.13585. 9811843 PMC24862

[91] Delaglio F, Grzesiek S, Vuister GW, Zhu G, Pfeifer J, Bax A 1995 NMRPipe: a multidimensional spectral processing system based on UniX pipes. J Biomol NMR 6:277–293. doi:10.1007/BF00197809. 8520220

[92] Konarev PV, Volkov VV, Sokolova AV, Koch MHJ, Svergun DI 2003 PRIMUS—a Windows-PC based system for small-angle scattering data analysis. J Appl Crystallogr 36:1277–1282. doi:10.1107/S0021889803012779.

[93] Svergun DI, Barberato C, Koch MHJ 1995 CRYSOL—a program to evaluate X-ray solution scattering of biological macromolecules from atomic coordinates. J Appl Crystallogr 28:768–773. doi:10.1107/S0021889895007047.

[94] Skubák P, Pannu NS 2013 Automatic protein structure solution from weak X-ray data. Nat Commun 4:2777. doi:10.1038/ncomms3777. 24231803 PMC3868232

[95] Winn MD, Ballard CC, Cowtan KD, Dodson EJ, Emsley P, Evans PR, Keegan RM, Krissinel EB, Leslie AG, McCoy A, McNicholas SJ, Murshudov GN, Pannu NS, Potterton EA, Powell HR, Read RJ, Vagin A, Wilson KS 2011 Overview of the CCP4 suite and current developments. Acta Crystallogr D Biol Crystallogr 67:235–242. doi:10.1107/S0907444910045749. 21460441 PMC3069738

[96] de Graaff RA, Hilge M, van der Plas JL, Abrahams JP 2001 Matrix methods for solving protein substructures of chlorine and sulfur from anomalous data. Acta Crystallogr D Biol Crystallogr 57:1857–1862. doi:10.1107/S0907444901016535. 11717499

[97] Abrahams JP, Leslie AG 1996 Methods used in the structure determination of bovine mitochondrial F1 ATPase. Acta Crystallogr D Biol Crystallogr 52:30–42. doi:10.1107/S0907444995008754. 15299723

[98] Adams PD, Afonine PV, Bunkóczi G, Chen VB, Davis IW, Echols N, Headd JJ, Hung LW, Kapral GJ, Grosse-Kunstleve RW, McCoy AJ, Moriarty NW, Oeffner R, Read RJ, Richardson DC, Richardson JS, Terwilliger TC, Zwart PH 2010 PHENIX: a comprehensive Python-based system for macromolecular structure solution. Acta Crystallogr D Biol Crystallogr 66:213–221. doi:10.1107/S0907444909052925. 20124702 PMC2815670

[99] Emsley P, Cowtan K 2004 Coot: model-building tools for molecular graphics. Acta Crystallogr D Biol Crystallogr 60:2126–2132. doi:10.1107/S0907444904019158. 15572765

[100] Skubák P, Murshudov GN, Pannu NS 2004 Direct incorporation of experimental phase information in model refinement. Acta Crystallogr D Biol Crystallogr 60:2196–2201. doi:10.1107/S0907444904019079. 15572772

[101] Brunger AT 2007 Version 1.2 of the crystallography and NMR system. Nat Protoc 2:2728–2733. doi:10.1038/nprot.2007.406. 18007608

[102] Brünger AT, Adams PD, Clore GM, DeLano WL, Gros P, Grosse-Kunstleve RW, Jiang JS, Kuszewski J, Nilges M, Pannu NS, Read RJ, Rice LM, Simonson T, Warren GL 1998 Crystallography and NMR system: a new software suite for macromolecular structure determination. Acta Crystallogr D Biol Crystallogr 54:905–921. doi:10.1107/S0907444998003254. 9757107

[103] Shen Y, Delaglio F, Cornilescu G, Bax A 2009 TALOS+: a hybrid method for predicting protein backbone torsion angles from NMR chemical shifts. J Biomol NMR 44:213–223. doi:10.1007/s10858-009-9333-z. 19548092 PMC2726990

[104] Sievers F, Wilm A, Dineen D, Gibson TJ, Karplus K, Li W, Lopez R, McWilliam H, Remmert M, Söding J, Thompson JD, Higgins DG 2011 Fast, scalable generation of high-quality protein multiple sequence alignments using Clustal Omega. Mol Syst Biol 7:539. doi:10.1038/msb.2011.75. 21988835 PMC3261699

[105] Cao B, Porollo A, Adamczak R, Jarrell M, Meller J 2006 Enhanced recognition of protein transmembrane domains with prediction-based structural profiles. Bioinformatics 22:303–309. doi:10.1093/bioinformatics/bti784. 16293670

[106] Vranken WF, Boucher W, Stevens TJ, Fogh RH, Pajon A, Llinas M, Ulrich EL, Markley JL, Ionides J, Laue ED 2005 The CCPN data model for NMR spectroscopy: development of a software pipeline. Proteins 59:687–696. doi:10.1002/prot.20449. 15815974

