# Supplementary figures and images for: Structural Basis for EarP-Mediated Arginine Glycosylation of Translation Elongation Factor EF-P
Source: mBio. 2017 Sep 26;8(5):e01412-17. doi: 10.1128/mBio.01412-17 (PMC5615199; doi:10.1128/mBio.01412-17)

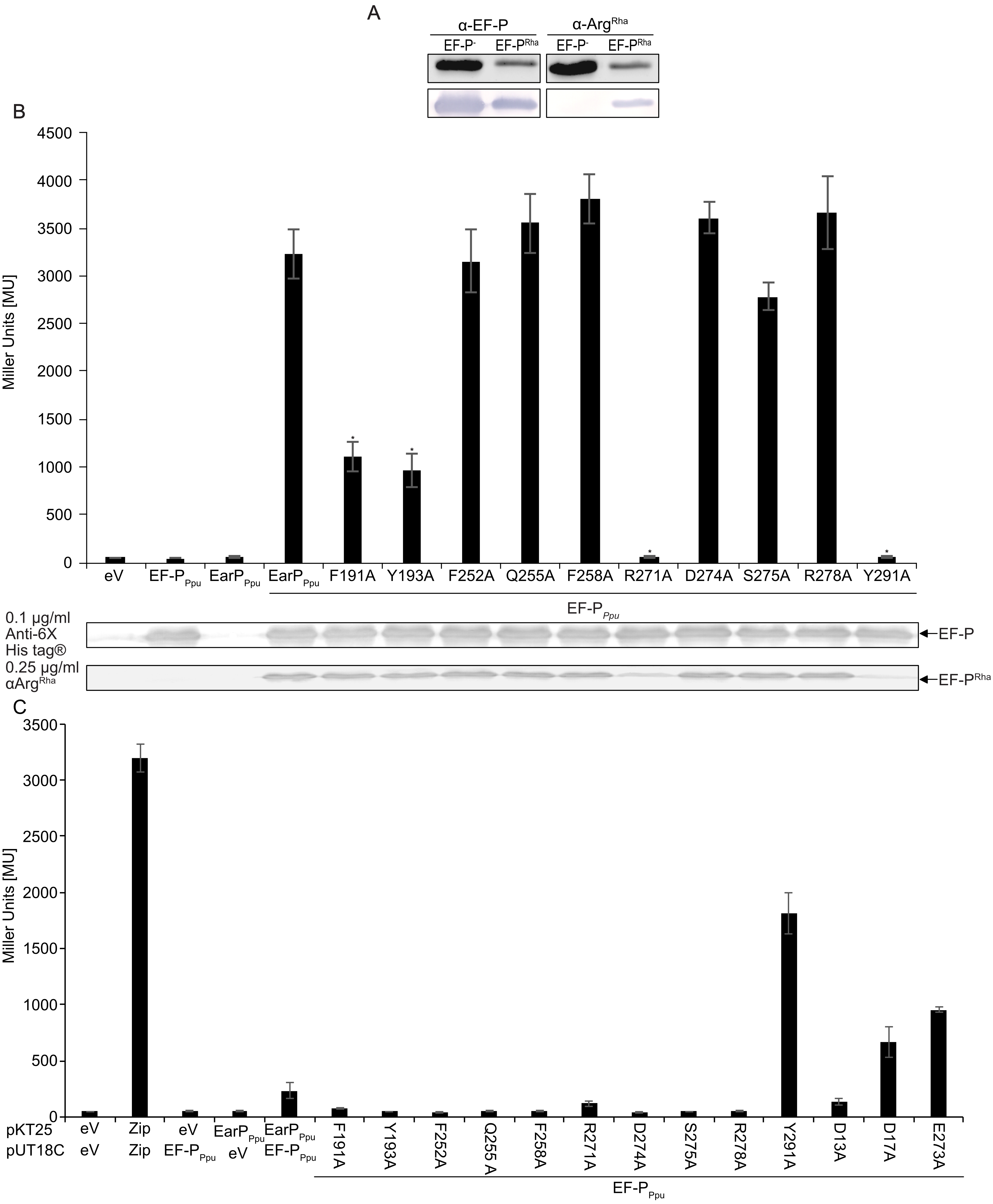

Supplement: FIG S1 [file mbo005173507sf1.tif]

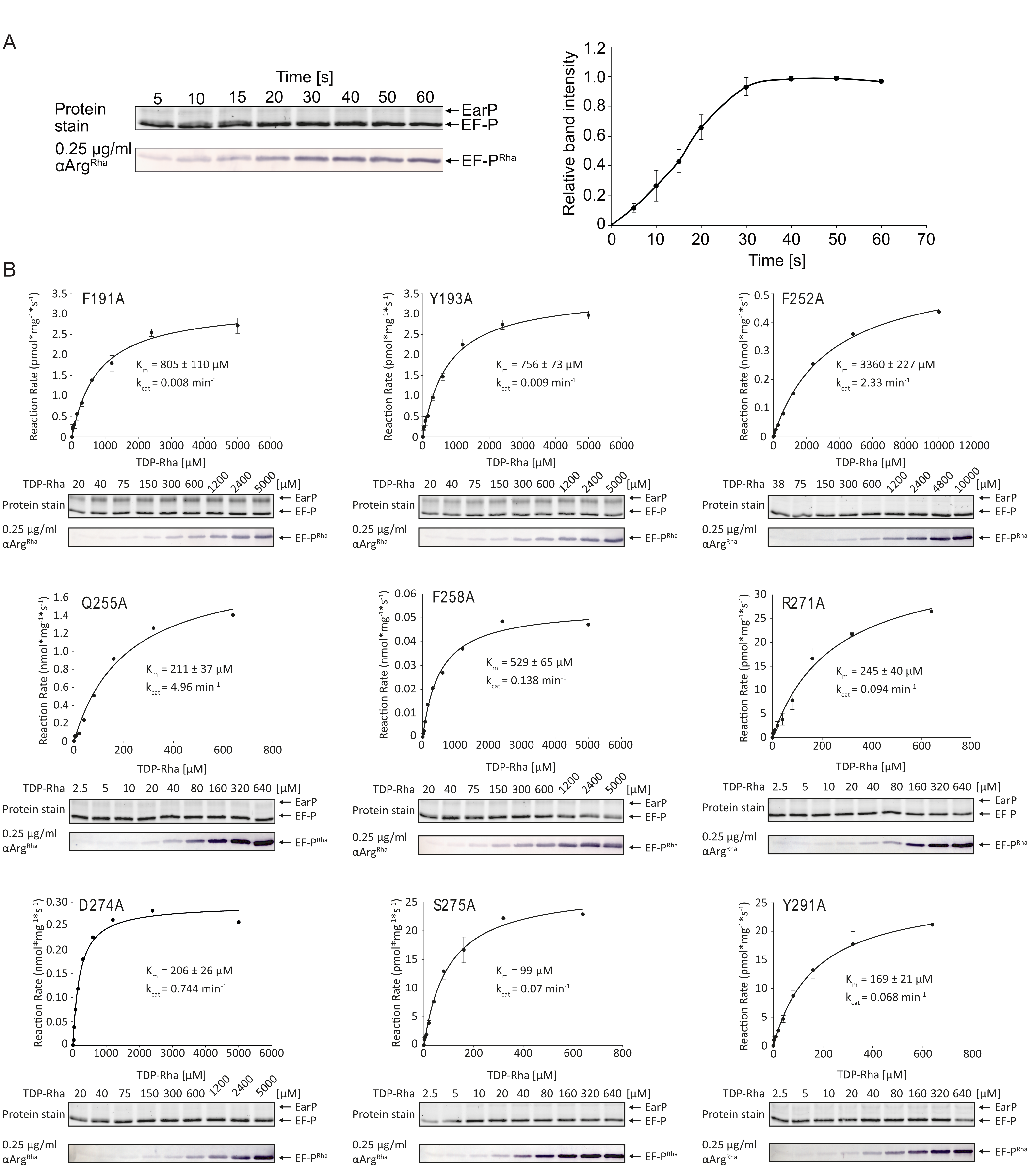

Supplement: FIG S2 [file mbo005173507sf2.tif]

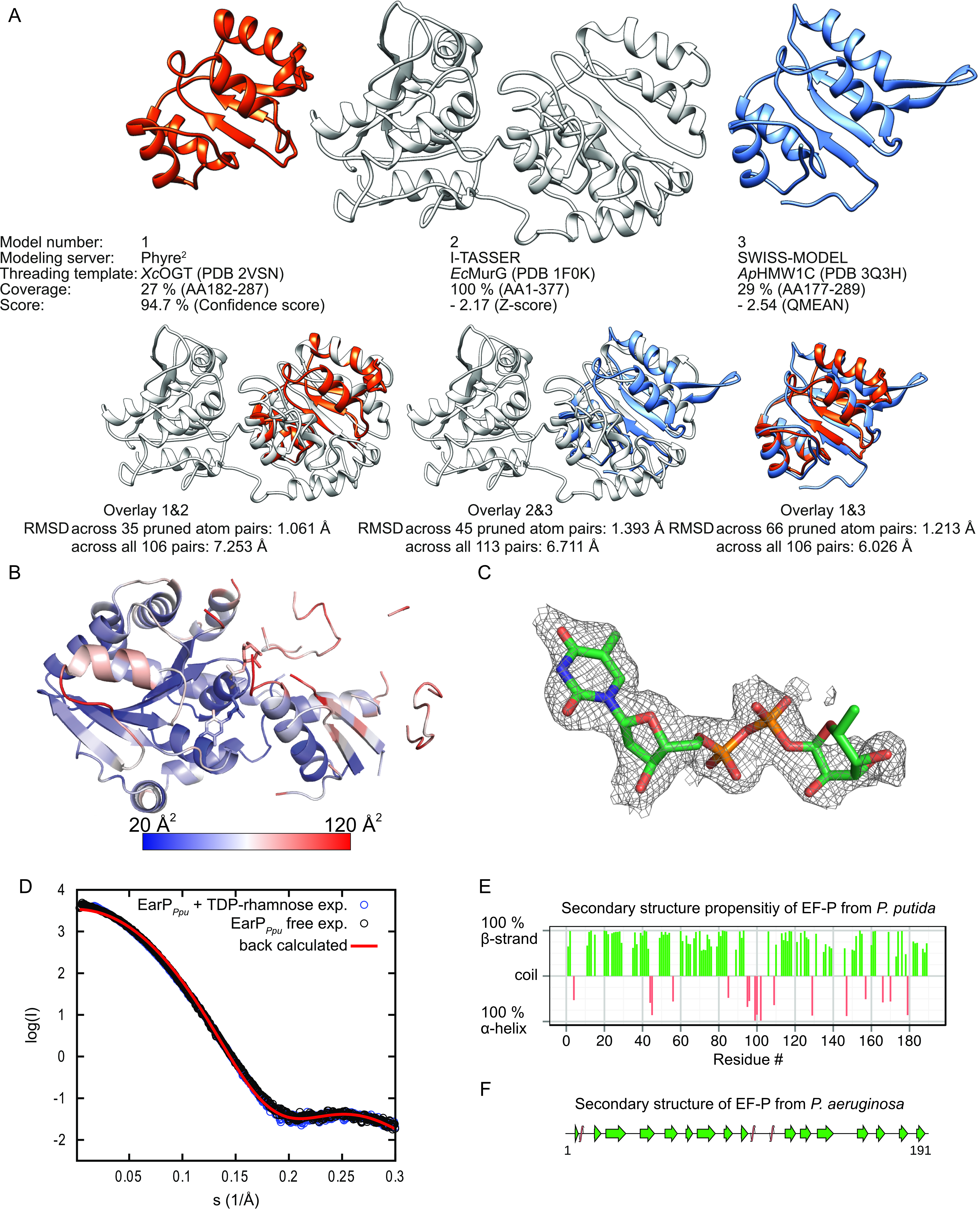

Supplement: FIG S3 [file mbo005173507sf3.tif]

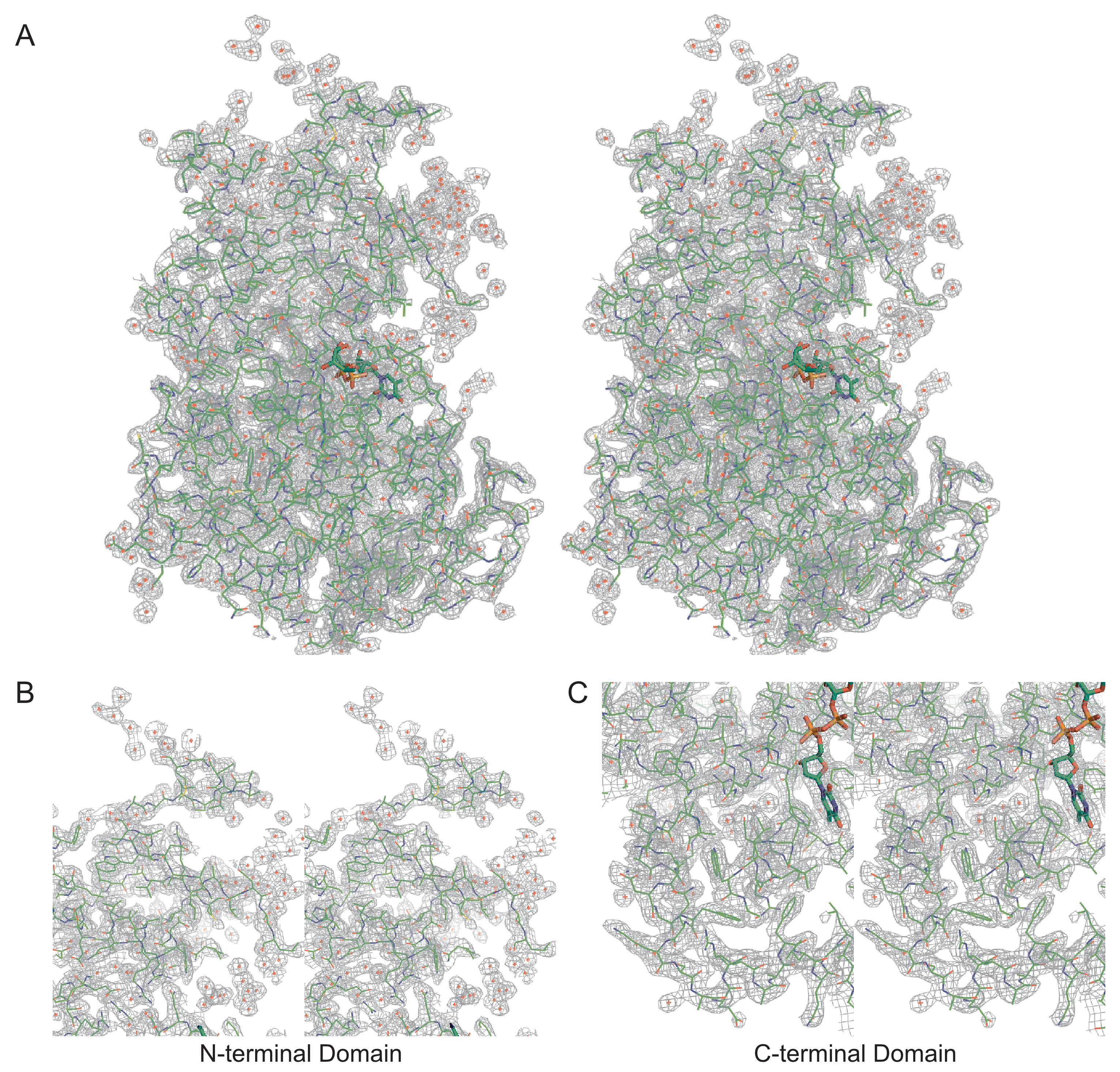

Supplement: FIG S4 [file mbo005173507sf4.tif]

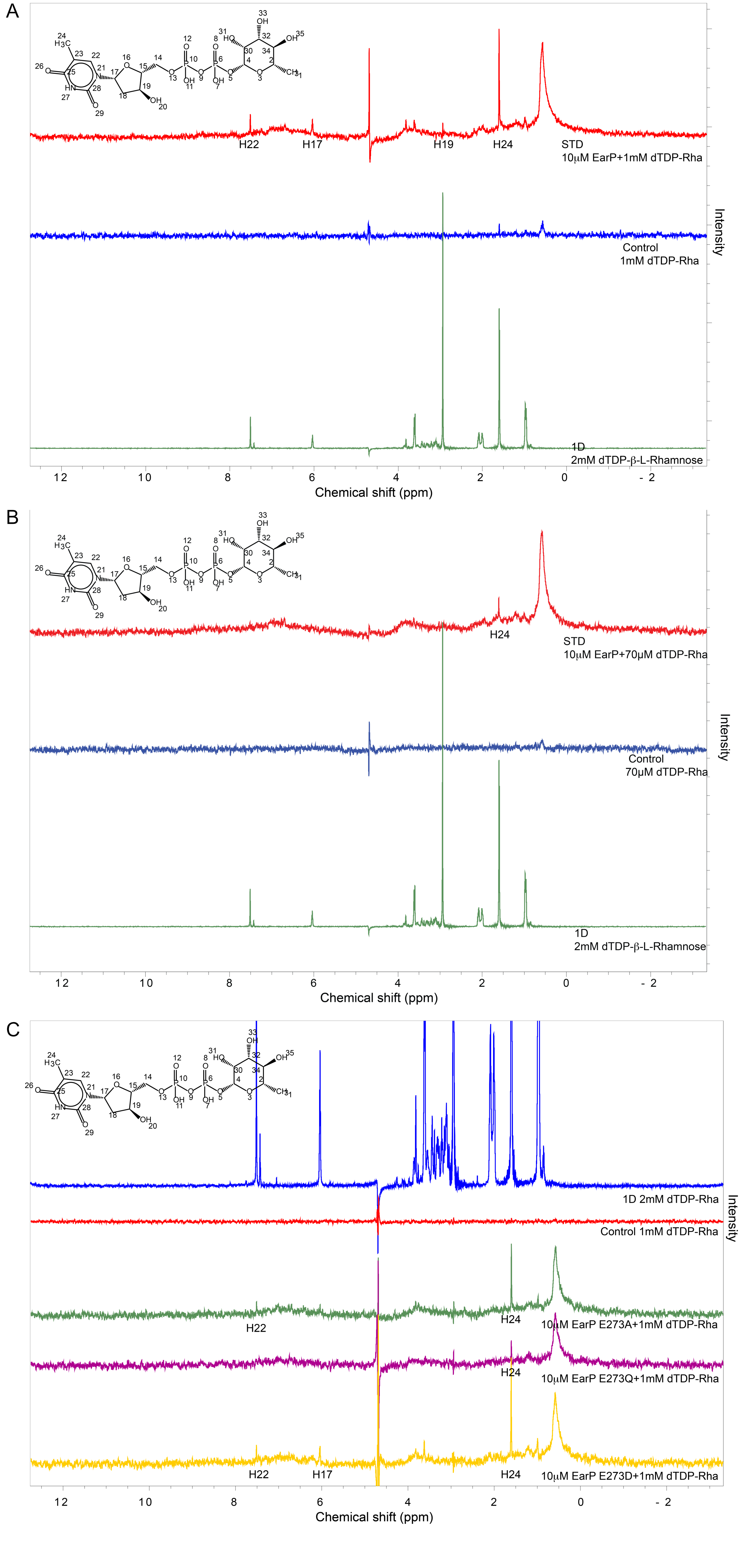

Supplement: FIG S5 [file mbo005173507sf5.tif]

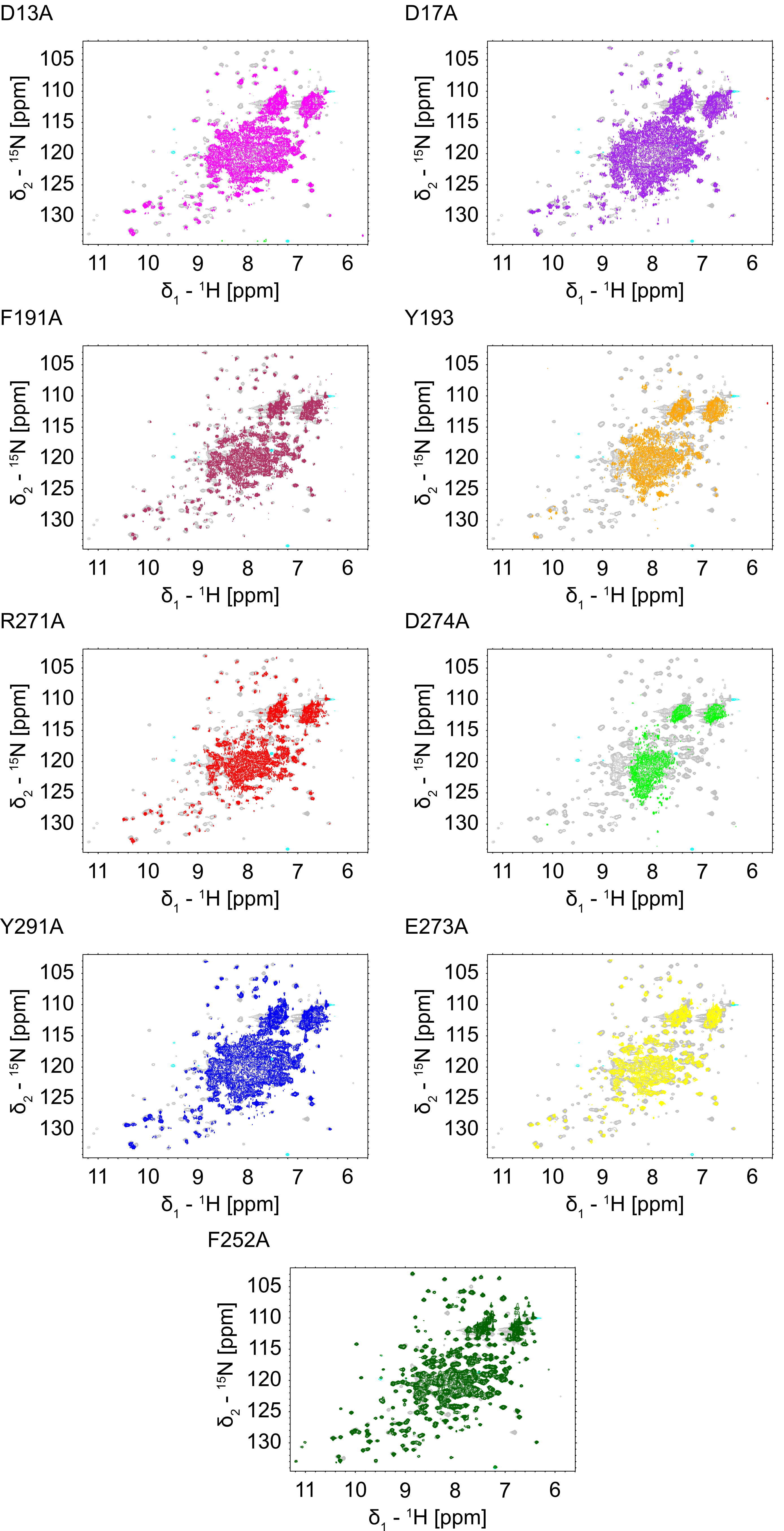

Supplement: FIG S7 [file mbo005173507sf7.tif]
